# Countrywide analysis of heat- and cold-related mortality trends in the Czech Republic: growing inequalities under recent climate warming

**DOI:** 10.1093/ije/dyad141

**Published:** 2023-10-19

**Authors:** Tomáš Janoš, Joan Ballester, Pavel Čupr, Hicham Achebak

**Affiliations:** RECETOX, Faculty of Science, Masaryk University, Brno, Czech Republic; ISGlobal, Barcelona, Spain; RECETOX, Faculty of Science, Masaryk University, Brno, Czech Republic; ISGlobal, Barcelona, Spain; Inserm, France Cohortes, Paris, France

**Keywords:** Heat, cold, climate change, temperature, mortality, adaptation, inequalities

## Abstract

**Background:**

Only little is known about trends in temperature–mortality associations among the most vulnerable subgroups, especially in the areas of central and eastern Europe, which are considered major climatic hotspots in terms of heatwave exposure. Thus, we aimed to assess trends in temperature-related mortality in the Czech Republic by sex, age and cause of death, and to quantify the temporal evolution of possible inequalities.

**Methods:**

We collected daily time series of all-cause (1987–2019) and cause-specific (1994–2019) mortality by sex and age category, and population-weighted daily mean 2-metre temperatures for each region of the Czech Republic. We applied a quasi-Poisson regression model to estimate the trends in region-specific temperature–mortality associations, with distributed lag non-linear models and multivariate random-effects meta-analysis to derive average associations across the country. We then calculated mortality attributable to non-optimal temperatures and implemented the indicator of sex- and age-dependent inequalities.

**Results:**

We observed a similar risk of mortality due to cold temperatures for men and women. Conversely, for warm temperatures, a higher risk was observed for women. Results by age showed a clear pattern of increasing risk due to non-optimum temperatures with increasing age category. The relative risk (RR) related to cold was considerably attenuated in most of the studied subgroups during the study period, whereas an increase in the RR associated with heat was seen in the overall population, in women, in the age category 90+ years and with respect to respiratory causes. Moreover, underlying sex- and age-dependent inequalities experienced substantial growth.

**Conclusions:**

Our findings suggest ongoing adaptation to cold temperatures. Mal/adaptation to hot temperatures occurred unequally among population subgroups and resulted in growing inequalities between the sexes and among age categories.

Key Messages

- First countrywide study of trends in temperature-related mortality in central and eastern Europe.
- We observed considerable reduction in cold-related risk of death.
- Maladaptation to heat existed in most of the population subgroups.
- Diverging trends in heat-related vulnerability exacerbated subgroup inequalities.

## Introduction

Climate change has already affected Europe in an unprecedented manner, resulting in a much steeper increase in average surface air temperatures compared with global figures^1^ and the hottest summer on record in 2022.^2^ Not only average surface air temperature, but also the frequency and intensity of heatwave events has increased rapidly in Europe, with the Czech Republic as one of the major hot spots.^3^

According to the Global Burden of Disease Study 2019, both high and low ambient temperatures are leading environmental risk factors.^4^ Generally, cold accounts for ∼10 times more deaths than heat^5^^,^^6^ but recent projections indicate the predominance of heat-attributable deaths during the second half of the twenty-first century in the absence of population adaptation to global warming.^6–8^ Nevertheless, these projections showed high regional differences across countries and offered no information on population subgroups.

Many studies have described temporal changes in heat-related vulnerability. The majority of them have reported the significant attenuation of risk during the last few decades, suggesting ongoing adaptation processes in the assessed populations,^9–16^ which could be driven by the pure physiological acclimatization response of the population and/or by non-climate factors such as socio-economic development and improvements in housing conditions and healthcare services.^12^^,^^17^ In contrast, despite the fact, that cold is responsible for a larger proportion of temperature-related deaths,^5^^,^^6^ only little is known about temporal variation in cold-related mortality, with no consistent conclusion.^9^^,^^12–14^^,^^16^^,^^18^

From a public health perspective, when assessing temperature-related risk and attributable mortality, stratified analysis is crucial for identifying the most susceptible population subgroups.^14^ The analysis of total mortality might hide diverging patterns between different population subgroups (by sex, age, cause of death), might thwart the identification of vulnerable ones and might thus complicate the possible implementation of actions aimed at mitigation and adaptation. Studies reporting temperature-related risk and/or temperature-related mortality associated with population subgroups have yielded heterogeneous results; moreover, stratified analysis is still missing in many regions, including the Czech Republic. Most studies have shown higher susceptibility to heat in women^11^^,^^16^^,^^18^ whereas men were more susceptible to cold.^13^^,^^14^^,^^16^^,^^19^ Increasing susceptibility to non-optimum temperatures with increasing age category was also observed,^14^^,^^18^^,^^20^ presumably due to age-related changes in the thermoregulation response.^21^ In contrast, some studies have reported the opposite in relation to cold exposure, particularly in age-specific response^16^^,^^19^^,^^22^ but also in sex-specific response.^18^^,^^23^

To date, only a limited number of studies have considered both temporal changes in relative risk (RR) and stratification by susceptible population subgroups (by sex and age). Results suggest no temporal trend in inequality.^11^^,^^13^^,^^14^^,^^18–20^ However, the quantification of sex- and age-dependent inequalities and their temporal evolution is missing. Furthermore, trends of heat- and cold-related mortality by cause of death, sex and age category have not yet been described for the Czech Republic, nor for the rest of central or eastern Europe. Understanding this issue at a country level, i.e. to cover both urban and rural areas, is critical with respect to supporting the implementation of mitigation and adaptation measures under present climate change,^24^ especially in countries that have experienced turbulent socio-economic changes in the last few decades. The Czech Republic has undergone steep development following political transition processes in central and eastern Europe. Investments in healthcare took place in parallel with improvements in life expectancy by 8.4 years in men and by 6.9 years in women^25^ accompanied by an almost 8% increase in the proportion of population aged 65+ years between 1987 and 2019.^26^ Economic growth accelerated rapidly and a marked expansion of higher education took place.^27^ However, the benefits of transformation have been unequally distributed with metropolitan areas on the one hand and old industrial regions, vast rural areas and regions located on borders on the other.^28^ In addition, the Czech Republic also lacks gender equality, with one of the highest disparities among the Member States of the European Union and basically no improvement between 2013 and 2019.^29^

Thus, here, we assessed spatial and temporal trends in heat- and cold-related mortality in the Czech Republic, which is the first countrywide study of its kind in the area of central and eastern Europe. In addition, we performed a stratified analysis by sex, age and cause of death in order to identify trends among vulnerable population subgroups. In so doing, we quantified inequalities and identified their spatial distribution and temporal evolution.

## Methods

### Data sources

Daily counts of all-cause (1987–2019) and cardiovascular and respiratory (1994–2019) mortality, disaggregated by sex, 15-year age categories (0–14, 15–29, …, 75–89, ≥90 years) and region of residence (14 units) were provided by the Institute of Health Information and Statistics of the Czech Republic. Only the age categories 60–74, 75–89 and 90 years and older were included in the analysis, as the number of daily deaths was large enough to guarantee model convergence. Population gridded (1 km^2^) data sets were obtained from Eurostat^30^ for the year 2006 as the representative year of the study period. Daily gridded (0.10° × 0.10°) observations of daily mean 2-metre temperatures (°C) were obtained from E-OBS (version 24.0e) of the European Climate Assessment and Dataset (ECA&D)^31^ and transformed into regional estimates by using the population-weighted average value for each region. Neither daily mortality nor temperature data had missing values.

### Statistical analysis

In the first step, we estimated the cause-, sex- and age-specific temperature–mortality associations for the whole study period and for data subsets of 13-year moving periods (to achieve at least two non-overlapping periods) in each of the 14 regions of the Czech Republic by using a generalized linear regression model with quasi-Poisson distribution. Specifically, the model included an intercept, a categorical variable of day of the week to take into account the weekly cycle in mortality, a natural cubic spline of time with 9 degrees of freedom (df) per year to control for seasonal and long-term trends in mortality and a cross-basis function of temperature produced by a distributed lag non-linear model to describe the non-linear and delayed associations between temperature and mortality.^32^ The exposure–response function of the cross basis was modelled with a natural cubic spline with three internal knots placed at the 10th, 75th and 90th percentiles of the daily temperature distribution for all-cause and cardiovascular mortality and with three internal knots placed at the 10th, 50th and 90th percentiles of the daily temperature distribution for respiratory mortality. Selection of the knots was based on a quasi-likelihood version of the Akaike information criterion (Q-AIC). The lag–response function of the cross basis was modelled with an intercept and three internal knots placed at equally spaced values in the log scale. The lag period was extended up to 21 days to capture the long-delayed effects of cold temperatures and potential short-term harvesting.

In the second step, we used multivariate random-effects meta-analysis^33^ for the whole study period and for all the data subsets of 13-year moving periods separately to pool the region-specific estimates obtained in the previous stage, thus obtaining the average associations across the country for the whole study period and for all the data subsets of the 13-year moving periods, which were reported as RR. We also used the meta-analysis to derive the best linear unbiased predictions in each region. The best linear unbiased prediction provides improved estimates of the temperature–mortality associations in each region and is also used to estimate the mortality burden attributable to temperatures in the following step.

In the third step, the RR associated with the temperature–mortality associations in each region were transformed into attributable mortality (i.e. attributable numbers and fractions of deaths due to heat and cold days) following the methodology described by Gasparrini and Leone.^34^ Heat and cold days were defined as days with temperatures higher and lower than the minimum mortality temperature and extreme heat and cold days as days warmer and colder than the 97.5th and 2.5th temperature percentiles of the whole study period, respectively. Finally, 95% empirical CIs of attributable mortality were calculated through Monte Carlo simulations assuming a multivariate normal distribution of the fixed-effect coefficients.

Finally, to quantify potential sex- and age-dependent inequalities in temperature-related risk and their temporal evolution, we implemented an inequality index. This indicator is calculated as the ratio between the RRs of compared population subgroups at specific temperature percentiles. An inequality index of 1 reflects perfect equality between two population subgroups; therefore, if it deviates from 1 over the course of time, inequalities are growing. Conversely, if it approaches 1, the inequality gap is becoming smaller.

The modelling choices were tested in several sensitivity analyses by varying the number of df per year used to control for the seasonal and long-term trends, the number and placement of knots in the exposure–response function and the number of lag days.

All statistical analyses and visualizations were performed using R software (version 4.1.1) using the packages *splines*, *dlnm* (for the first-stage regression), *mixmeta* (for the second-stage multivariate random-effects meta-analysis), *modifiedmk* (for modified versions of Mann–Kendall trend test) and *tmap*.

## Results

**Figure 1.**
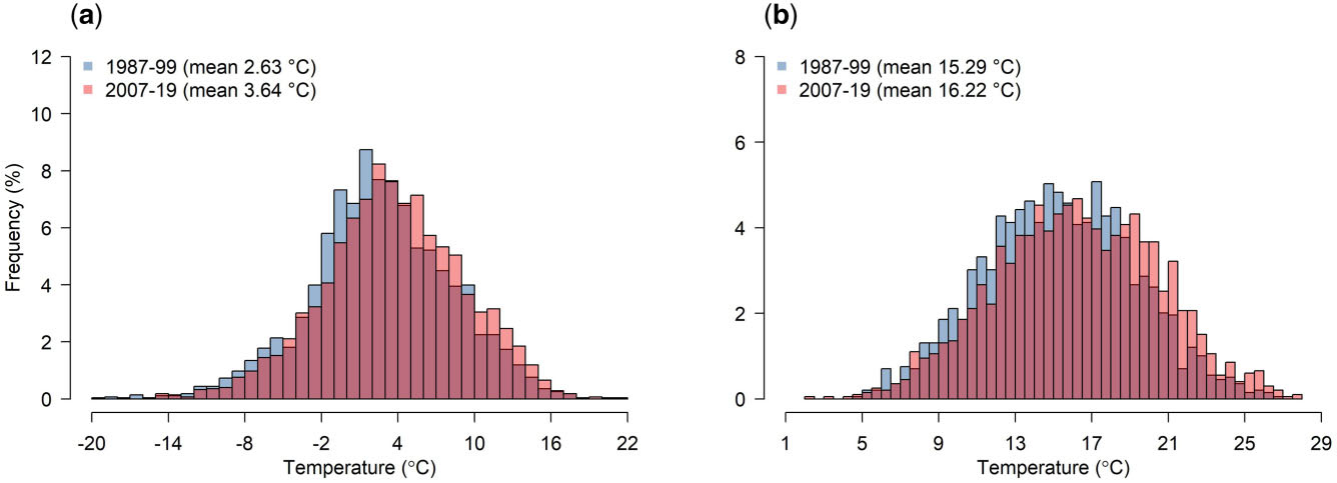
Distribution of daily mean temperatures between (a) October and April and (b) May and September in the Czech Republic between 1987 and 1999 and between 2007 and 2019

We collected data from 14 regions covering the whole of the territory of the Czech Republic and 3 717 972 deaths from all causes were recorded (women comprising 49.5% of cases) during the study period (1987–2019). Individuals in the age category 75–89 years accounted for most of the mortality (45.3%, of which women comprised 59% of cases), followed by age category 60–74 years (30.8%, of which women comprised 39.1% of cases) and 90+ years (7.8%, of which women comprised 74.4% of cases). The data set also included 1 434 490 cardiovascular-related deaths (women comprising 54.4% of cases) and 151 333 respiratory-related deaths (women comprising 45.4% of cases), which represented 50.4% and 5.3% of total mortality, respectively (for region-specific numbers and descriptive statistics, see Supplementary Tables S1–S4, available as Supplementary data at *IJE* online). Over the course of the study, the daily mean temperature distributions shifted towards warmer temperatures, with an average increase of 1°C observed between the periods 1987–99 and 2007–19 in both cold and hot months (from 2.63°C to 3.64°C in October–April and from 15.29°C to 16.22°C in May–September) (Figure 1 and Supplementary Figure S1, available as Supplementary data at *IJE* online).

**Figure 2.**
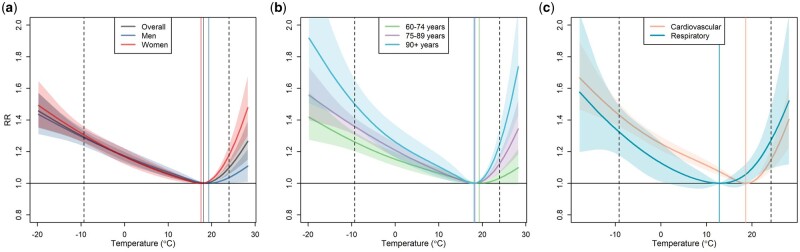
Temperature–mortality associations from the model for the whole study period stratified by (a) sex, (b) age category and (c) cause of death. Dashed vertical lines denote the 1st and 99th percentiles of the study period-specific temperature distribution. Solid lines denote subgroup-specific minimum mortality temperatures. RR,  relative risk

The overall temperature–mortality associations obtained for the whole study period stratified by sex, age category and cause of death are presented in Figure 2 (for region-specific exposure–response functions and the geographical distribution of the regions of the Czech Republic, see Supplementary Figures S1–S9, available as Supplementary data at *IJE* online). All the exposure–response curves had a ‘hockey stick’ shape, with a steeper increase in warm temperatures compared with cold temperatures. The only exception was respiratory mortality, which exhibited a ‘U’-shaped curve and a minimum mortality temperature shifted towards lower temperatures. Results by sex showed a higher risk of mortality due to hot temperatures in women and no sex difference for cold temperatures. Using age stratification, we found a clear pattern of increasing risk due to non-optimum temperatures with increasing age category.

Hot and cold temperatures were responsible for >9000 deaths per year during the study course, which was 8.35% (95% empirical CI: 7.22–9.48) of all deaths recorded (Tables 1 and 2). The attributable fraction (AF) and attributable number (AN) of deaths due to cold were generally larger for all population subgroups. Considering days with extreme and moderate temperatures, there were more deaths on days with extreme heat than on days with moderate heat. In contrast, the cold AN and AF of deaths were 10 times larger on days with moderate cold than on days with extreme cold. A different pattern was observed in the case of respiratory mortality, where the contribution of extreme temperatures was comparable but we observed a slight shift from moderate cold to moderate heat. This is largely explained by the symmetry of the curve (Figure 2) and the shift in the minimum mortality temperature towards lower temperatures compared with cardiovascular and all-cause mortality (Tables 1 and 2). Consistent results by sex and age were observed, as in the temperature-related RR of death, with higher AF and AN of deaths due to heat in women, comparable AF and AN of deaths due to cold and increasing relative impacts of non-optimum temperatures with increasing age category.

**Table 1.** Cold- and heat-attributable fraction of deaths with 95% empirical confidence intervals from the model for the whole study period

	Minimum mortality temperature (°C)	Minimum mortality temperature percentile		Cold days		Heat days	
			Total	**Extreme cold** ^a^	**Moderate cold** ^b^	**Moderate heat** ^b^	**Extreme heat** ^a^
Overall	18.1	87	8.35% (7.22–9.48)	0.64% (0.61–0.67)	7.34% (6.10–8.34)	0.14% (0.11–0.16)	0.23% (0.18–0.27)
By sex							
Women	17.6	85	8.45% (7.31–9.53)	0.67% (0.63–0.71)	7.14% (6.02–8.18)	0.28% (0.23–0.32)	0.36% (0.30–0.41)
Men	19.3	91	8.29% (6.95–9.47)	0.62% (0.57–0.65)	7.55% (6.42–8.69)	0.03% (0.01–0.05)	0.09% (0.05–0.13)
By age							
60–74 years	19.3	91	7.61% (6.91–8.28)	0.57% (0.54–0.60)	6.94% (6.29–7.55)	0.02% (0.01–0.03)	0.07% (0.05–0.10)
75–89 years	18.1	87	9.85% (9.17–10.52)	0.75% (0.72–0.79)	8.64% (7.95–9.33)	0.17% (0.13–0.22)	0.28% (0.22–0.33)
90+ years	18.1	87	12.9% (10.8–14.5)	0.92% (0.85–0.99)	11.1% (9.03–12.8)	0.33% (0.26–0.39)	0.53% (0.47–0.58)
By cause							
Cardiovascular	18.6	88	12.4% (11.8–13.1)	0.89% (0.84–0.93)	11.0% (10.4–11.6)	0.17% (0.12–0.21)	0.31% (0.24–0.37)
Respiratory	12.8	65	8.39% (5.59–10.8)	0.83% (0.71–0.93)	5.61% (3.20–7.66)	1.45% (0.64–2.21)	0.50% (0.37–0.60)

a Extreme cold and heat defined as temperatures lower than the 2.5th location-specific percentile (extreme cold) and higher than the 97.5th location-specific percentile (extreme heat).

b Moderate cold and heat defined as temperatures lower than the minimum mortality temperature and higher than the 2.5th location-specific percentile (moderate cold) and higher than the minimum mortality temperature and lower than the 97.5th location-specific percentile (moderate heat).

**Table 2.** Cold- and heat-attributable number of deaths per year with 95% empirical confidence intervals from the model for the whole study period

	Minimum mortality temperature (°C)	Minimum mortality temperature percentile		Cold days		Heat days	
			Total	**Extreme cold** ^a^	**Moderate cold** ^b^	**Moderate heat** ^b^	**Extreme heat** ^a^
Overall	18.1	87	9403 (8140–10 678)	725 (690–756)	8272 (6868–9399)	153 (123–183)	253 (203–304)
By sex							
Women	17.6	85	4711 (4075–5314)	375 (353–394)	3981 (3359–4562)	154 (129–178)	201 (169–231)
Men	19.3	91	4715 (3952–5387)	350 (327–371)	4297 (3650–4945)	18 (7–28)	50 (27–72)
By age							
60–74 years	19.3	91	2640 (2399–2873)	200 (189–209)	2407 (2181–2620)	8 (4–12)	25 (17–34)
75–89 years	18.1	87	5027 (4681–5368)	385 (369–401)	4410 (4085–4763)	88 (67–110)	143 (112–170)
90+ years	18.1	87	1135 (954–1280)	82 (75–87)	977 (797–1127)	29 (23–35)	47 (42–51)
By cause							
Cardiovascular	18.6	88	6844 (6487–7221)	492 (463–515)	60 190 (5757–6393)	91 (66–114)	171 (134–202)
Respiratory	12.8	65	488 (326–626)	48 (41–54)	326 (187–446)	84 (37–129)	29 (21–35)

a Extreme cold and heat defined as temperatures lower than the 2.5th location-specific percentile (extreme cold) and higher than the 97.5th location-specific percentile (extreme heat).

b Moderate cold and heat defined as temperatures lower than the minimum mortality temperature and higher than the 2.5th location-specific percentile (moderate cold) and higher than the minimum mortality temperature and lower than the 97.5th location-specific percentile (moderate heat).

**Figure 3.**
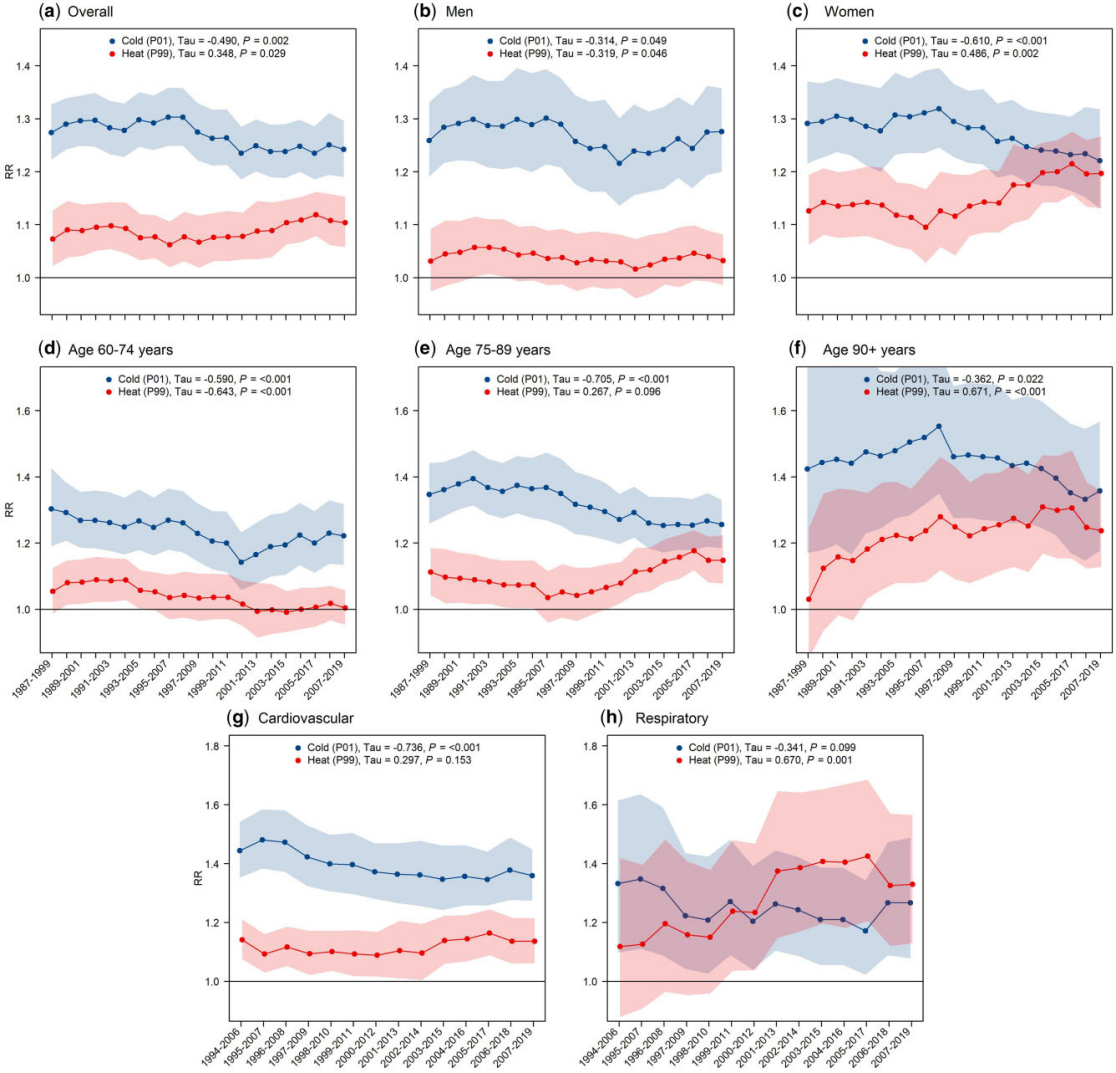
Relative risk of death at the 1st (cold) and 99th (heat) temperature percentiles of the whole study period from the model with subsets of 13-year moving periods. Mann–Kendall’s tau value and *P*-value from the Nonparametric Block Bootstrapped Mann–Kendall Trend Test. RR, relative risk; P01, temperature percentile 1; P99, temperature percentile 99

The RR related to cold was (*P*-value < 0.05, Mann–Kendall Trend Test) attenuated in the majority of the studied subgroups during the study period or did not experience any evolution (respiratory mortality). Diverse trends could be observed in the case of exposure to heat. A reduction in the RR associated with heat was seen only in the age category 60–74 years and men. The RR of death in the age category 75–89 years and in cardiovascular diseases remained stable (no important increase/decrease) during the study period and we did not notice any signs of adaptation to hot temperatures. Moreover, the RR corresponding to the 99th temperature percentile increased considerably in the overall population, in women, in the age category 90+ years and in respiratory disease mortality (Figure 3; region-specific curves are presented in Supplementary Figures S10–S17, available as Supplementary data at *IJE* online).

**Figure 4.**
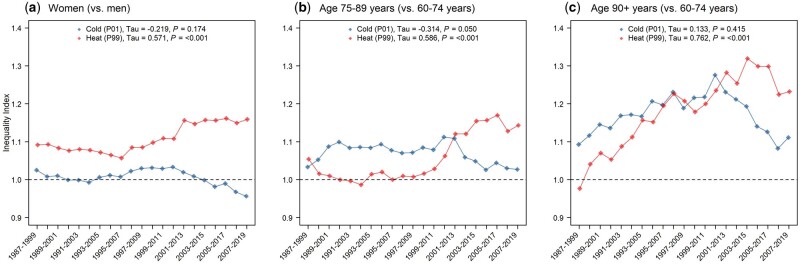
Evolution of inequality index at the 1st (cold) and 99th (heat) temperature percentiles during the study period. Mann–Kendall’s tau value and *P*-value from the Nonparametric Block Bootstrapped Mann–Kendall Trend Test. P01, temperature percentile 1; P99, temperature percentile 99

RRs derived from the whole study period models showed larger inequalities related to heat (an inequality index of 1.14 for women vs men, 1.09 for the age category 75–89 vs 60–74 years and 1.21 for the age category 90+ vs 60–74 years) when compared with cold (inequality indices 1.01, 1.08 and 1.19, respectively). More importantly, trends in heat-related inequality indices showed increasing inequalities during the study period (*P*-value < 0.05, Mann–Kendall Trend Test). Conversely, cold-related inequalities remained stable (Figure 4).

**Figure 5.**
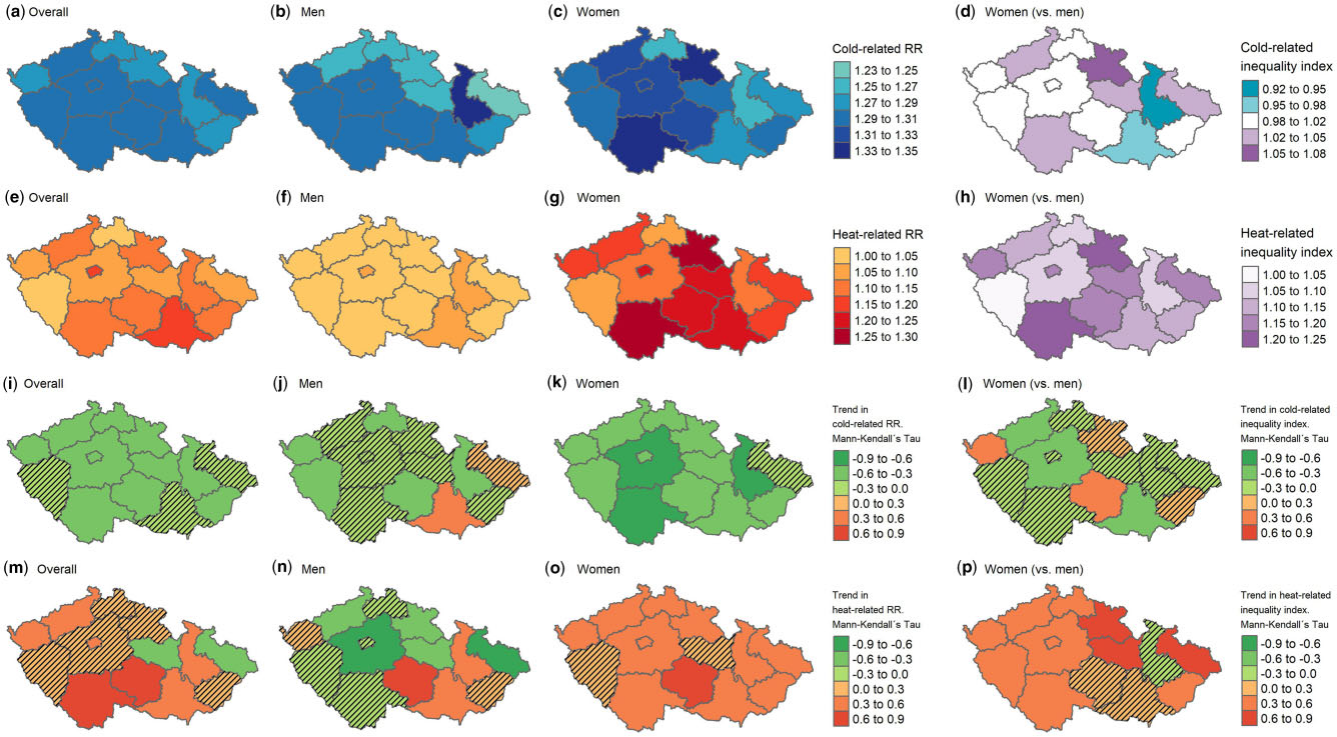
Spatial variation in relative risks and the inequality index at the 1st (cold) and 99th (heat) temperature percentiles by sex and their temporal trends during the study period. Mann–Kendall’s tau value and *P*-value from the Nonparametric Block Bootstrapped Mann–Kendall Trend Test. Hatched regions are not significant. RR, relative risk; TP01, temperature percentile 1; TP99, temperature percentile 99

**Figure 6.**
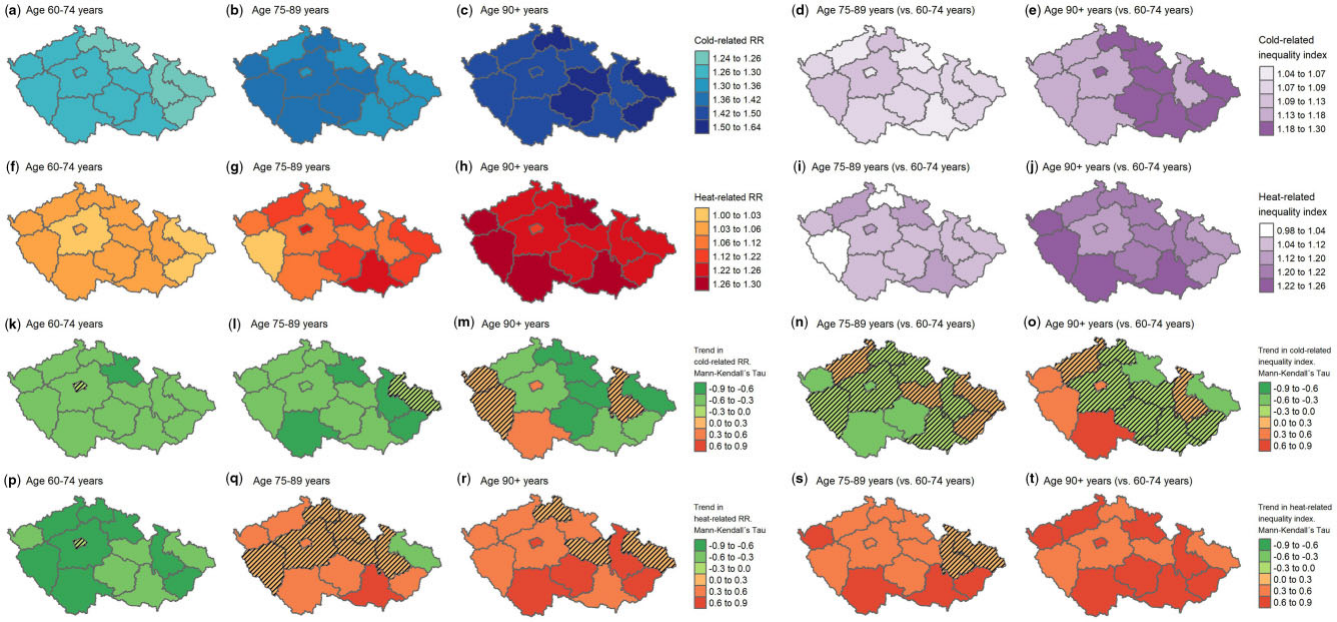
Spatial variation in relative risks and the inequality index at the 1st (cold) and 99th (heat) temperature percentiles by age category and temporal trends during the study period. Mann–Kendall’s tau value and *P*-value from the Nonparametric Block Bootstrapped Mann–Kendall Trend Test. Hatched regions are not significant. RR, relative risk; TP01, temperature percentile 1; TP99, temperature percentile 99

The spatial variability in RRs and inequalities at the 1st (cold) and 99th (heat) temperature percentiles and their temporal evolution can be seen in Figures 5 and 6 for both sexes and age categories, respectively. The maps show high spatial variability in RRs and their trends at both cold and hot temperatures across regions. For instance, the RR for the overall population at the 99th temperature percentile decreased during the study period in two regions whereas, in seven regions, a significant increase was recorded (see Map M; Figure 5). The same was also true for sex- and age-dependent inequalities and their temporal evolution. Although, in some of the regions, the inequality index was close to 1 (perfect equality between two population subgroups), in others it was >1.2 (Map H; Figure 5). Trends in sex- and age-dependent inequalities in relation to cold differed across regions (Map L; Figure 5 and Maps N and O; Figure 6), with a stable degree of inequality in most of them. A clearer pattern can be seen in trends of heat-related inequalities (Map P; Figure 5 and Maps S and T; Figure 6). In the vast majority of regions, growth was revealed, more prominent in inequalities by age.

Multiple sensitivity analyses suggested that the reported results were generally not dependent on modelling assumptions (see Supplementary Table S5, available as Supplementary data at *IJE* online).

## Discussion

To the best of our knowledge, this is the first countrywide study in the area of central and eastern Europe to assess temporal variations of temperature-related mortality. Furthermore, sex-, age- and cause of death-specific analyses allowed us to quantify trends in inequalities for the first time. We found similar vulnerability to cold temperatures for both sexes. Conversely, for warm temperatures, higher risk was observed for women. Our results also showed a considerable attenuation of risk related to cold temperatures whereas the pattern for hot temperatures was not so clear. Meanwhile, the divergent evolution of heat-related RRs strongly indicated growing sex- and age-related inequalities. Finally, our findings suggest substantial spatial variability in trends of mortality associated with non-optimum temperatures, but especially in inequalities and their evolution. These findings strongly support the need for countrywide research to cover differences in regional socio-economic and demographic status as well as in their rural, sub-urban or urban natures.^24^

The underlying cause of observed sex-dependent vulnerability may be physiological, behavioural or socio-economic. Women usually have a larger ratio of body surface to body mass and greater subcutaneous fat content, and show hormone-, menstrual cycle- and menopause-related fluctuations in body temperature and in some thermoregulatory processes,^35^^,^^36^ which might lead to greater vulnerability to the effects of heat.^37^ Nonetheless, the suggested biological difference in the sweating response of women seems to be an unlikely reason for their greater susceptibility to heat, except in unusual circumstances where the need to sweat is extreme.^35^^,^^38^ Not only biological issues, but also physical and social isolation and a worse financial status, which are more prevalent in elderly women, have been associated with increased mortality associated with hot temperatures.^18^^,^^37^ On the other hand, sex differences in cold appear to be less prominent than in heat. Although higher susceptibility to heat in women was reported in several studies,^11^^,^^14^^,^^16^^,^^18^^,^^19^^,^^39^ there is no consensus on outcomes linked to cold-related mortality, including studies proposing higher risk in women,^18^ in men^16^ and also no sex difference at all.^14^^,^^19^^,^^39^^,^^40^

With regard to age categories, the results for heat are consistent with other studies in which older individuals are considered as a population subgroup that is more susceptible to heat exposure.^14^^,^^16^^,^^18^^,^^20^^,^^33^^,^^35^ Otherwise, in the case of cold, evidence is not so straightforward as some studies showed higher^18^^,^^20^^,^^41^ or lower^16^ risk in older age categories or the pattern differed between the sexes.^14^^,^^39^ There are diverse mechanisms that could lead to increased vulnerability. Namely, ageing is associated with impaired thermoregulation processes and the presence of comorbidities.^21^ Higher vulnerability among the elderly may also relate to housing quality and social factors^18^^,^^41^ such as social isolation.^42^

With respect to stratification by cause of death, the influence of heat on respiratory mortality was greater than on mortality due to cardiovascular causes. Conversely, cold temperatures had a greater impact on cardiovascular mortality. The heat-related cause-specific pattern is in agreement with previous literature^11^^,^^43–47^ and potentially points towards the higher vulnerability of people with certain respiratory diseases already present when exposed to heat.^11^ Cause-specific cold-related effects are more difficult to discuss in view of the scant literature on this topic or its inconsistent findings.^43^^,^^45^^,^^46^

Estimated AF and AN of deaths are in good agreement with another multi-country study in Europe with the observational period between 1998 and 2012, where 7.80% and 0.38% of deaths in the Czech Republic were attributed to cold and heat exposure.^6^ One of the causes of the different attributions to heat and cold is that the minimum mortality temperature at which the risk is lowest was well above the third temperature quartile in the majority of our study subgroups and thus cold included the most days in the series. Nevertheless, when considering the contribution of extreme cold and heat days, the difference is reduced, which might be alarming as the occurrence and intensity of extreme heat events are still increasing;^3^ thus, the heat AF of deaths is expected to predominate during the second half of the twenty-first century.^6^^,^^7^

In light of recent climate warming, the observed adaptation to cold temperatures is rather the result of non-climate factors such as socio-economic development, housing conditions or improved healthcare services.^11^^,^^12^ The Czech Republic has experienced turbulent socio-economic changes in the few last decades due to the collapse of communism in 1989. The gross domestic product increased by 805% from 1990 to 2021.^48^ During the study period, life expectancy at birth grew by 8.4 years (from 67.9 years in 1987 to 76.3 years in 2019) in men and by 6.9 years (from 75.2 years in 1987 to 82.1 years in 2019) in women.^25^ However, this extension is accompanied by population ageing and thus increasing the population at risk, which might lead to amplification of temperature-related deaths.^20^ During the same period, the infant mortality rate, as a good indicator of the quality of healthcare services and the development of various societies,^49^ decreased from 12% to 2.6%.^25^ Regrettably, the evidence suggests that the general improvement has been unequally distributed in terms of growing regional disparities^28^ or gender equality^29^ as, for instance, the pension gender gap almost tripled during the last three decades.^50^ A similar substantial decrease in the risk of cold-related mortality was observed in Japan^13^ and in Spain in the case of all-cause mortality^16^ but also in cause-specific mortality.^14^^,^^19^ By contrast, in other regions of the USA, Canada, Sweden, the UK and South Korea, no clear pattern of cold-mortality temporal variation has been recognized^9^^,^^12^ or the trend has even been found to be increasing in Turin, Italy,^18^ pointing to inconclusive evidence with high spatial variability and high dependency on region-specific characteristics.

When considering adaptation to heat, the physiological acclimatization of the population to a changing climate also has to be taken into account.^12^ The presence of adaptation and a corresponding decrease in vulnerability to heat has already been recognized in a majority of studies in different settings.^9^^,^^11^^,^^12^^,^^16^ In our study, the results for warm temperatures are consistent with those only relating to the age category 60–74 years and men. In the remaining population subgroups, or with respect to cause-specific mortality, the trend was opposite or remained stable. These findings confirm the need for stratified analysis in order to identify the most susceptible population subgroups. Furthermore, it should be noted that divergent trends in heat-related risk of death emphasize the presence of inequalities among population subgroups.

The identified sex- and age-dependent inequalities and their possible rationale are discussed above. In addition, we did not find any clear trend in cold-related inequalities among sexes and age categories. This evidence thus suggests that non-climate adaptation factors, as the main drivers of the decrease in vulnerability to cold temperatures,^12^ improved similarly among population subgroups. Surprisingly, heat-related inequalities between the sexes grew significantly during the study period and, moreover, the increase was even steeper when comparing the age categories 75–89 and 90+ years with the age category 60–74 years.

Given the presence of stable cold-related inequalities, these seem rather to be a result of slower or insufficient physiological adaptation processes in the population subgroups that were already disadvantaged. The underlying physiological mechanism is not well understood but current evidence suggests that the capacity to adapt to heat is similar between the sexes; however, there are differences in the time course, where women may require a prolonged period of adaptation.^36^ Similarly, there is evidence showing that older individuals are capable of adapting, though the response to heat is attenuated after repeated exposure compared with younger individuals.^51^ This could indicate the possible more frequent exhaustion of physiological adaptation mechanisms and explain the observed increase in inequalities as exposure to heat occurs more often. The aforementioned differences in adaptation capacity by sex and age support the hypothesis that climate change exacerbates inequalities even between sexes and age categories within the same geographical area. Despite the fact that the opposite trends in cold- and heat-related inequalities point to some degree to the impairment physiological adaptation, the main emphasis should still be placed on socio-economic adaptation, which will play a major role in determining temperature-related excess mortality in the future and could be promoted through adaptation policies such as effective early warning systems, green spaces, investments in health systems and sustainable heating and cooling strategies.^52^

The region-specific findings support the notion that temperature-related mortality is highly determined by regional differences in socio-economic and demographic status. For instance, a recent study found that proportion of elderly people (80+ years) explains a significant fraction of the spatial heterogeneity.^53^ According to the OECD, the Czech Republic faces stark regional disparities across different dimensions, with the starkest disparities in terms of civic engagement, income, community and health.^54^ As in our study, Urban and colleagues,^55^ focusing on heat-related mortality in urban areas of Prague, the capital of the Czech Republic, also found an increasing risk of heat-related mortality compared with previous decades. However, it is noteworthy that this trend is mostly driven by women and older age categories and that the urbanized area of Prague is generally very different from other more rural regions. Accordingly, we can assume that these findings cannot easily be extrapolated to other geographical areas and population subgroups, as various factors driving changes in susceptibility are highly heterogeneous and locations deviate from each other. From this perspective, planned adaptation policies and interventions should be tuned to specific locations, such as urban areas or the most deprived ones, and populations at the highest risk, such as women and elderly people, rather than implemented generally.^24^

There are some notable limitations of our study. First, in our models, we were not able to adjust for air pollution or influenza epidemics due to data unavailability, nor did we control for relative humidity. Nevertheless, current literature shows that these factors would have had either no or only a minimal confounding impact on the temperature–mortality relationship.^43^^,^^56–59^ Second, we could not assess temperature–mortality associations, impacts, trends or inequalities by cross combining sex, age category and cause of death due to a lack of cases and thus a lack of statistical power.

## Conclusion

To conclude, we observed several differences in temperature-related mortality among population subgroups defined by sex, age and cause of death, with a higher risk due to cold in cardiovascular diseases, a similar risk due to cold in men and women, and a higher risk due to warm temperatures in women and respiratory diseases. Vulnerability to both non-optimal temperatures increased with increasing age category. Decreasing trends in cold-related RRs suggest ongoing adaptation to low temperatures. In contrast, divergent trends in heat-related risk among population subgroups even amplified the identified inequalities. Despite signs of the impairment of physiological adaptation, the main emphasis should still be placed on socio-economic adaptation, such as effective early warning systems, green spaces, investment in health systems and sustainable heating and cooling strategies. Additionally, through the observed regional variability, this study also demonstrated the need for planned adaptation policies and interventions that are more suitable for specific locations and populations rather than designed for general implementation.

## Ethics approval

The data used are anonymized and aggregated, and thus do not include any personal data. Therefore, ethics approval is not applicable.

## Supplementary Material

dyad141_Supplementary_Data

## Data Availability

The mortality data underlying this article were provided by the Institute of Health Information and Statistics of the Czech Republic (https://www.uzis.cz/index-en.php) by permission. Data will be shared on request to the corresponding author with permission of the Institute of Health Information and Statistics of the Czech Republic. The temperature data underlying this article are available in an E-OBS data set from the EU-FP6 project UERRA at http://www.uerra.eu. The underlying population data were obtained from Eurostat (https://ec.europa.eu/eurostat/web/main/home). All the data used in this study are routinely collected and contain no information about specific people. Mortality data can be requested through the Institute of Health Information and Statistics of the Czech Republic (https://www.uzis.cz/index-en.php). Temperature data could be obtained from the E-OBS data set from the EU-FP6 project UERRA (http://www.uerra.eu) and underlying population distribution data from Eurostat (https://ec.europa.eu/eurostat/web/gisco/geodata/reference-data/population-distribution-demography/geostat).
