# Supplementary material for: Countrywide analysis of heat- and cold-related mortality trends in the Czech Republic: growing inequalities under recent climate warming
Source: Int J Epidemiol. 2023 Oct 19;53(1):dyad141. doi: 10.1093/ije/dyad141 (PMC10859142; doi:10.1093/ije/dyad141)
Supplement: dyad141_Supplementary_Data [file dyad141_supplementary_data.pdf]

Supplementary information to

Countrywide analysis of heat- and cold-related mortality trends in the Czech Republic: growing inequalities under recent climate warming

Authors:

Tomáš Janoš[1], Joan Ballester[2], Pavel Čupr[1]*, Hicham Achebak[2-3]

Affiliations:

[1] RECETOX, Faculty of Science, Masaryk University, Kotlarska 2, Brno, Czech Republic

[2] ISGlobal, Barcelona, Spain

[3] Inserm, France Cohortes, Paris, France

*Corresponding author: Pavel Čupr, RECETOX Centre, Faculty of Science, Masaryk University, Kamenice 753/5, pavilion A29, 625 00 Brno, Czech Republic, Phone number: +420 549 493 511, E-mail: pavel.cupr@recetox.muni.cz

Supplementary Figure S1 Spatial distribution of average daily mean temperatures between 1987 and 2019 between October and April (A) and between May and September (B) in the Czech Republic. Differences in average daily mean temperatures between 1987 and 1999 and 2007 and 2019 between October and April (C) and between May and September (D) in the Czech Republic.

Supplementary Table S1 Descriptive statistics on numbers of deaths in the regions of the Czech Republic (all-cause mortality, 1987-2019)

Supplementary Table S2 Relative numbers (percentage of total) of deaths in the regions of the Czech Republic (all-cause mortality, 1987-2019)

Supplementary Table S3 Descriptive statistics on numbers of deaths in the regions of the Czech Republic (cause-specific mortality, 1994-2019)

Supplementary Table S4 Relative numbers (percentage of total) of deaths in the regions of the Czech Republic (cause-specific mortality, 1987-2019)

Supplementary Figure S2 Temperature-mortality associations among overall population in the regions of the Czech Republic from model for whole study period. RR=relative risk. Dashed vertical lines denote the 1st and 99th percentiles of the specific temperature distribution. Solid lines denote specific MMT.

Supplementary Figure S3 Temperature-mortality associations among men population in the regions of the Czech Republic from model for whole study period. RR=relative risk. Dashed vertical lines denote the 1st and 99th percentiles of the specific temperature distribution. Solid lines denote specific MMT.

Supplementary Figure S4 Temperature-mortality associations among women population in the regions of the Czech Republic from model for whole study period. RR=relative risk. Dashed vertical lines denote the 1st and 99th percentiles of the specific temperature distribution. Solid lines denote specific MMT.

Supplementary Figure S5 Temperature-mortality associations among population aged 60-74 in the regions of the Czech Republic from model for whole study period. RR=relative risk. Dashed vertical lines denote the 1st and 99th percentiles of the specific temperature distribution. Solid lines denote specific MMT.

Supplementary Figure S6 Temperature-mortality associations among population aged 75-89 in the regions of the Czech Republic from model for whole study period. RR=relative risk. Dashed vertical lines denote the 1st and 99th percentiles of the specific temperature distribution. Solid lines denote specific MMT.

Supplementary Figure S7 Temperature-mortality associations among population aged 90+ in the regions of the Czech Republic from model for whole study period. RR=relative risk. Dashed vertical lines denote the 1st and 99th percentiles of the specific temperature distribution. Solid lines denote specific MMT.

Supplementary Figure S8 Temperature-mortality associations from cardiovascular diseases among overall population in the regions of the Czech Republic from model for whole study period. RR=relative risk. Dashed vertical lines denote the 1st and 99th percentiles of the specific temperature distribution. Solid lines denote specific MMT.

Supplementary Figure S9 Temperature-mortality associations from respiratory diseases among overall population in the regions of the Czech Republic from model for whole study period. RR=relative risk. Dashed vertical lines denote the 1st and 99th percentiles of the specific temperature distribution. Solid lines denote specific MMT.

Supplementary Figure S10 Trend in RR of death at 1st (cold) and 99th (heat) temperature percentile among overall population in the regions of the Czech Republic from model with subsets of 13-year moving periods. RR=relative risk, TP01=temperature percentile 1, TP99=temperature percentile 99.

Supplementary Figure S11 Trend in RR at 1st (cold) and 99th (heat) temperature percentile among men population in the regions of the Czech Republic from model with subsets of 13-year moving periods. RR=relative risk, TP01=temperature percentile 1, TP99=temperature percentile 99.

Supplementary Figure S12 Trend in RR of death at 1st (cold) and 99th (heat) temperature percentile among women population in the regions of the Czech Republic from model with subsets of 13-year moving periods. RR=relative risk, TP01=temperature percentile 1, TP99=temperature percentile 99.

Supplementary Figure S13 Trend in RR of death at 1st (cold) and 99th (heat) temperature percentile among population aged 60-74 in the regions of the Czech Republic from model with subsets of 13-year moving periods. RR=relative risk, TP01=temperature percentile 1, TP99=temperature percentile 99.

Supplementary Figure S14 Trend in RR of death at 1st (cold) and 99th (heat) temperature percentile among population aged 75-89 population in the regions of the Czech Republic from model with subsets of 13-year moving periods. RR=relative risk, TP01=temperature percentile 1, TP99=temperature percentile 99.

Supplementary Figure S15 Trend in RR of death at 1st (cold) and 99th (heat) temperature percentile among population aged 90+ in the regions of the Czech Republic from model with subsets of 13-year moving periods. RR=relative risk, TP01=temperature percentile 1, TP99=temperature percentile 99.

Supplementary Figure S16 Trend in RR of death from cardiovascular diseases at 1st (cold) and 99th (heat) temperature percentile among overall population in the regions of the Czech Republic from model with subsets of 13-year moving periods. RR=relative risk, TP01=temperature percentile 1, TP99=temperature percentile 99.

Supplementary Figure S17 Trend in RR of death from respiratory diseases at 1st (cold) and 99th (heat) temperature percentile among overall population in the regions of the Czech Republic from model with subsets of 13-year moving periods. RR=relative risk, TP01=temperature percentile 1, TP99=temperature percentile 99.

Supplementary Table S5 Sensitivity analysis by varying modelling choices – Attributable fraction (%) for total, cold and heat temperatures for whole study period for different parameters in the fitting of the temperature-mortality relationship (95% empirical CI).

Supplementary Figure S1 Spatial distribution of average daily mean temperatures between 1987 and 2019 between October and April (A) and between May and September (B) in the Czech Republic. Differences in average daily mean temperatures between 1987 and 1999 and 2007 and 2019 between October and April (C) and between May and September (D) in the Czech Republic.

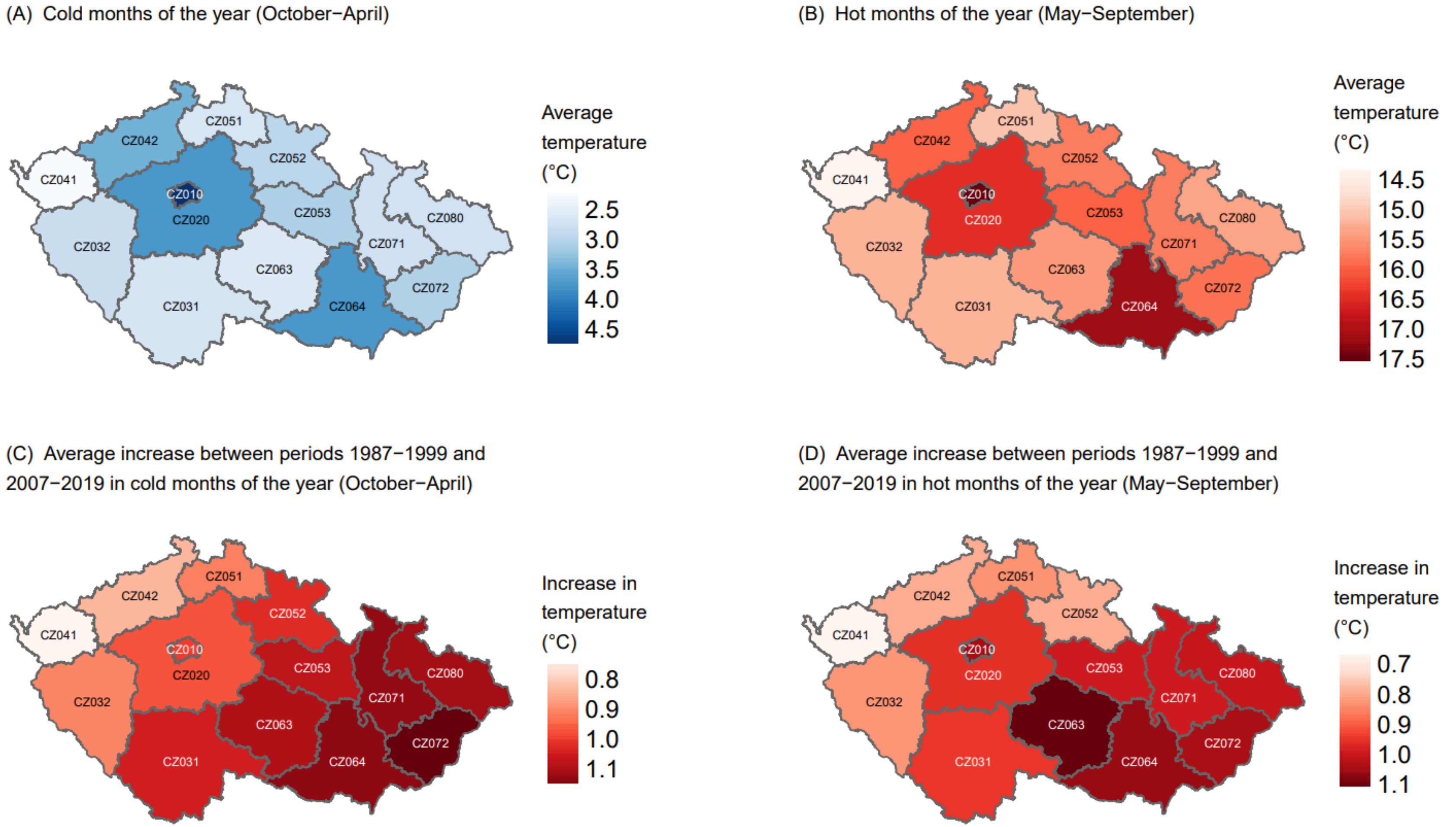

Regions (NUTS3): CZ010 Hlavni mesto Praha, CZ020 Stredocesky kraj, CZ031 Jihocesky kraj, CZ032 Plzensky kraj, CZ041 Karlovarsky kraj, CZ042 Ustecky kraj, CZ051 Liberecky kraj, CZ052 Kralovehradecky kraj, CZ053 Pardubicky kraj, CZ063 Kraj Vysocina, CZ064 Jihomoravsky kraj, CZ071 Olomoucky kraj, CZ072 Zlinsky kraj, CZ080 Moravskoslezsky kraj.

Supplementary Table S1 Descriptive statistics on numbers of deaths in the regions of the Czech Republic (all-cause mortality, 1987-2019)

Region	Population (2021 census)	Deaths per day mean (min-max)	Overall population			Age 60-74			Age 75-89			Age 90+		
			Total	Women	Men	Total	Women	Men	Total	Women	Men	Total	Women	Men
CZ010	1,301,432	37.2 (13-79)	448,684	235,798	212,886	128,755	54,051	74,704	215,702	128,901	86,801	42,580	31,482	11,098
CZ020	1,415,463	36.6 (14-75)	441,433	219,646	221,787	137,247	52,928	84,319	204,196	121,344	82,852	33,354	25,159	8,195
CZ031	631,803	18.4 (4-40)	221,536	108,959	112,577	67,441	25,742	41,699	103,241	60,256	42,985	17,129	12,769	4,360
CZ032	581,436	17.3 (3-43)	208,386	103,472	104,914	65,354	25,496	39,858	97,719	57,474	40,245	14,746	10,979	3,767
CZ041	279,103	8.5 (1-29)	102,495	48,963	53,532	36,381	14,551	21,830	40,140	23,627	16,513	5,564	4,211	1,353
CZ042	789,098	25.2 (6-54)	303,418	146,906	156,512	105,288	42,198	63,090	123,197	73,745	49,452	17,371	13,249	4,122
CZ051	435,220	12.4 (1-32)	149,620	73,529	76,091	47,398	18,729	28,669	65,567	38,653	26,914	11,275	8,375	2,900
CZ052	538,303	16.6 (4-39)	199,564	100,365	99,199	58,781	23,060	35,721	95,029	55,554	39,475	17,539	12,935	4,604
CZ053	510,037	15.1 (2-41)	181,470	89,864	91,606	54,389	21,006	33,383	85,513	50,126	35,387	14,560	10,668	3,892
CZ063	497,661	14.8 (2-34)	178,669	87,180	91,489	51,401	19,264	32,137	86,237	49,345	36,892	15,361	11,139	4,222
CZ064	1,197,651	33.8 (13-69)	407,048	202,923	204,125	118,236	45,516	72,720	189,221	110,930	78,291	37,199	27,779	9,420
CZ071	619,788	18.8 (4-48)	226,209	110,758	115,451	68,090	26,181	41,909	102,120	59,804	42,316	18,236	13,431	4,805
CZ072	564,331	17.3 (4.39)	208,571	100,612	107,959	62,093	23,360	38,733	95,028	55,404	39,624	16,518	12,070	4,448
CZ080	1,162,841	36.6 (14-92)	440,869	211,532	229,337	144,316	55,524	88,792	181,435	108,100	73,335	29,886	22,630	7,256

Supplementary Table S2 Relative numbers (percentage of total) of deaths in the regions of the Czech Republic (all-cause mortality, 1987-2019)

Region	Population (2021 census)	Overall population			Age 60-74			Age 75-89			Age 90+		
		Total	Women	Men	Total	Women	Men	Total	Women	Men	Total	Women	Men
CZ010	1,301,432	100%	53%	47%	29%	12%	17%	48%	29%	19%	9%	7%	2%
CZ020	1,415,463	100%	50%	50%	31%	12%	19%	46%	27%	19%	8%	6%	2%
CZ031	631,803	100%	49%	51%	30%	12%	19%	47%	27%	19%	8%	6%	2%
CZ032	581,436	100%	50%	50%	31%	12%	19%	47%	28%	19%	7%	5%	2%
CZ041	279,103	100%	48%	52%	35%	14%	21%	39%	23%	16%	5%	4%	1%
CZ042	789,098	100%	48%	52%	35%	14%	21%	41%	24%	16%	6%	4%	1%
CZ051	435,220	100%	49%	51%	32%	13%	19%	44%	26%	18%	8%	6%	2%
CZ052	538,303	100%	50%	50%	29%	12%	18%	48%	28%	20%	9%	6%	2%
CZ053	510,037	100%	50%	50%	30%	12%	18%	47%	28%	20%	8%	6%	2%
CZ063	497,661	100%	49%	51%	29%	11%	18%	48%	28%	21%	9%	6%	2%
CZ064	1,197,651	100%	50%	50%	29%	11%	18%	46%	27%	19%	9%	7%	2%
CZ071	619,788	100%	49%	51%	30%	12%	19%	45%	26%	19%	8%	6%	2%
CZ072	564,331	100%	48%	52%	30%	11%	19%	46%	27%	19%	8%	6%	2%
CZ080	1,162,841	100%	48%	52%	33%	13%	20%	41%	25%	17%	7%	5%	2%

Supplementary Table S3 Descriptive statistics on numbers of deaths in the regions of the Czech Republic (cause-specific mortality, 1994-2019)

Region	Population (2021 census)	Cardiovascular			Respiratory		
		Total	Women	Men	Total	Women	Men
CZ010	1,301,432	165,602	93,740	71,862	17,273	8,389	8,884
CZ020	1,415,463	171,690	94,270	77,420	16,252	7,288	8,964
CZ031	631,803	81,661	44,152	37,509	10,420	4,852	5,568
CZ032	581,436	77,084	41,859	35,225	8,605	3,947	4,658
CZ041	279,103	36,845	19,336	17,509	4,894	2,229	2,665
CZ042	789,098	115,615	61,725	53,890	11,240	4,931	6,309
CZ051	435,220	57,321	31,033	26,288	5,357	2,502	2,855
CZ052	538,303	78,801	43,176	35,625	8,711	4,066	4,645
CZ053	510,037	70,096	37,851	32,245	8,754	3,985	4,769
CZ063	497,661	72,173	38,642	33,531	7,163	3,220	3,943
CZ064	1,197,651	162,448	89,593	72,855	15,375	7,026	8,349
CZ071	619,788	88,172	47,511	40,661	9,318	4,149	5,169
CZ072	564,331	86,022	46,236	39,786	7,484	3,319	4,165
CZ080	1,162,841	170,960	91,640	79,320	20,487	8,798	11,689

Supplementary Table S4 Relative numbers (percentage of total) of deaths in the regions of the Czech Republic (cause-specific mortality, 1987-2019)

Region	Population (2021 census)	Cardiovascular			Respiratory		
		Total	Women	Men	Total	Women	Men
CZ010	1,301,432	42%	24%	18%	4%	2%	2%
CZ020	1,415,463	43%	24%	19%	4%	2%	2%
CZ031	631,803	41%	22%	19%	5%	2%	3%
CZ032	581,436	42%	23%	19%	5%	2%	3%
CZ041	279,103	41%	21%	19%	5%	2%	3%
CZ042	789,098	43%	23%	20%	4%	2%	2%
CZ051	435,220	43%	23%	19%	4%	2%	2%
CZ052	538,303	42%	23%	19%	5%	2%	3%
CZ053	510,037	42%	22%	19%	5%	2%	3%
CZ063	497,661	43%	23%	20%	4%	2%	2%
CZ064	1,197,651	43%	24%	19%	4%	2%	2%
CZ071	619,788	42%	23%	19%	4%	2%	2%
CZ072	564,331	43%	23%	20%	4%	2%	2%
CZ080	1,162,841	42%	22%	19%	5%	2%	3%

Supplementary Figure S2 Temperature-mortality associations among overall population in the regions of the Czech Republic from model for whole study period. RR=relative risk. Dashed vertical lines denote the 1st and 99th percentiles of the specific temperature distribution. Solid lines denote specific MMT.

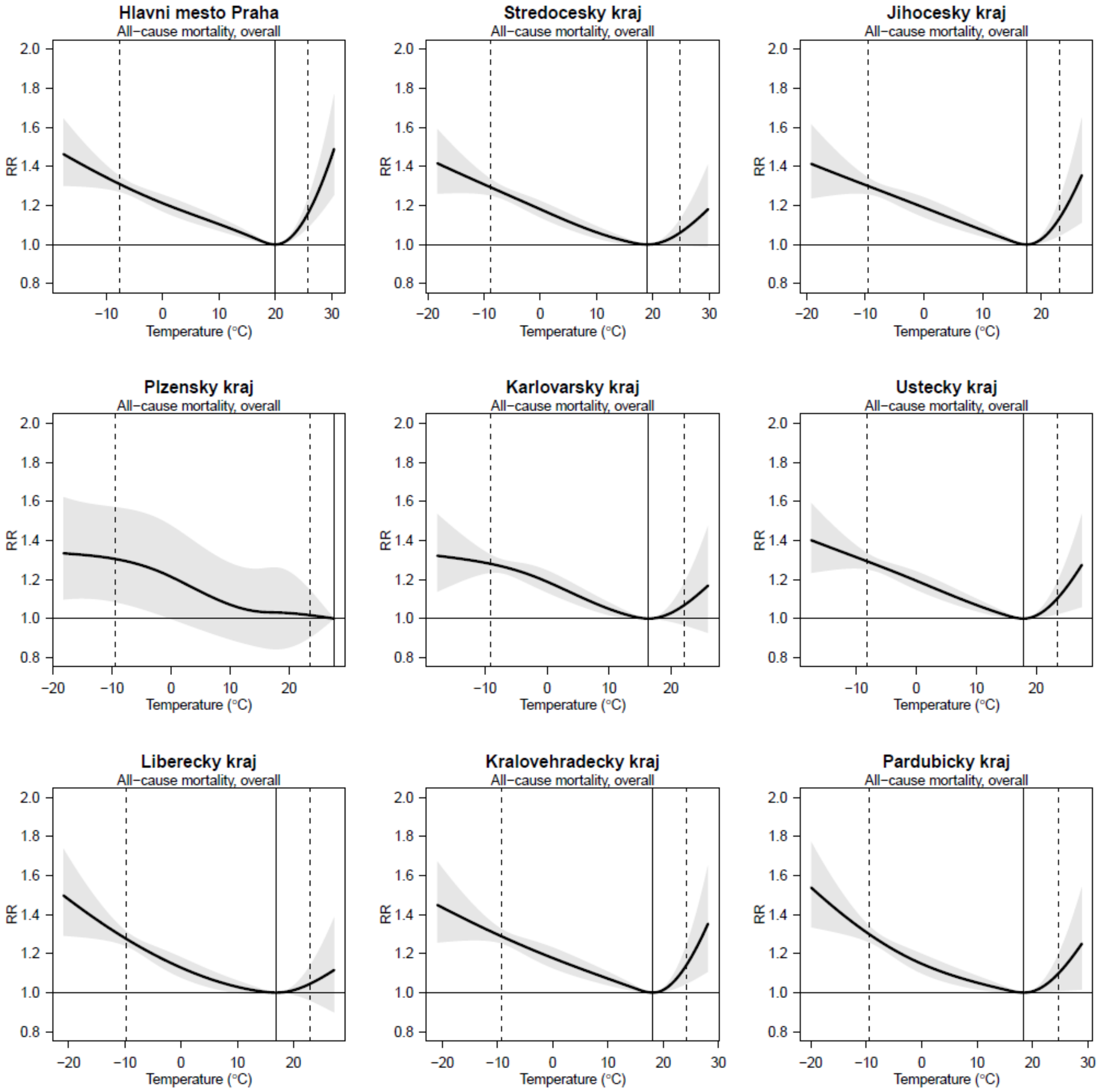

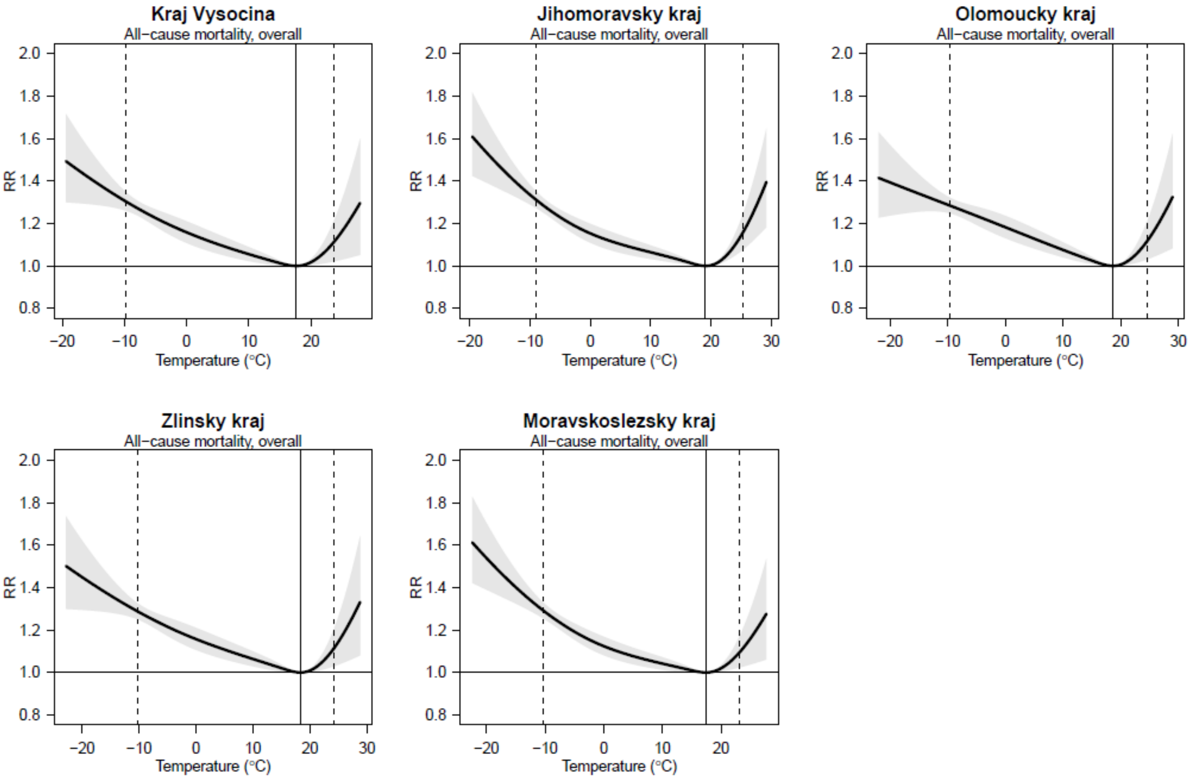
Kraj Vysocina
All−cause mortality, overall
Jihomoravsky kraj
All−cause mortality, overall
Olomoucky kraj
All−cause mortality, overall
Zlinsky kraj
All−cause mortality, overall
Moravskoslezsky kraj
All−cause mortality, overall
RR
Temperature (°C)
2.0
1.8
1.6
1.4
1.2
1.0
0.8
−20
−10
0
10
20
30

Supplementary Figure S3 Temperature-mortality associations among men population in the regions of the Czech Republic from model for whole study period. RR=relative risk. Dashed vertical lines denote the 1st and 99th percentiles of the specific temperature distribution. Solid lines denote specific MMT.

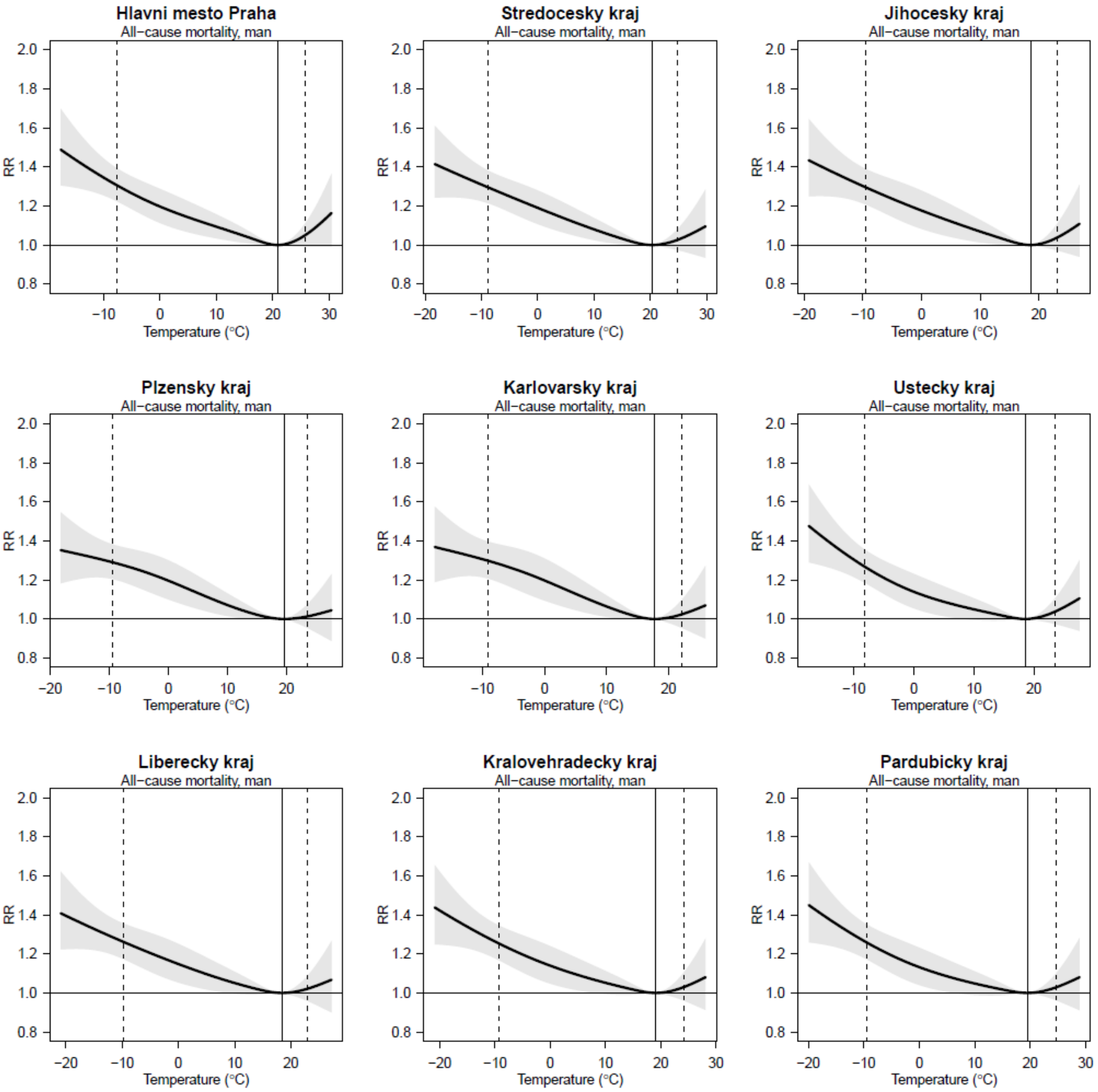

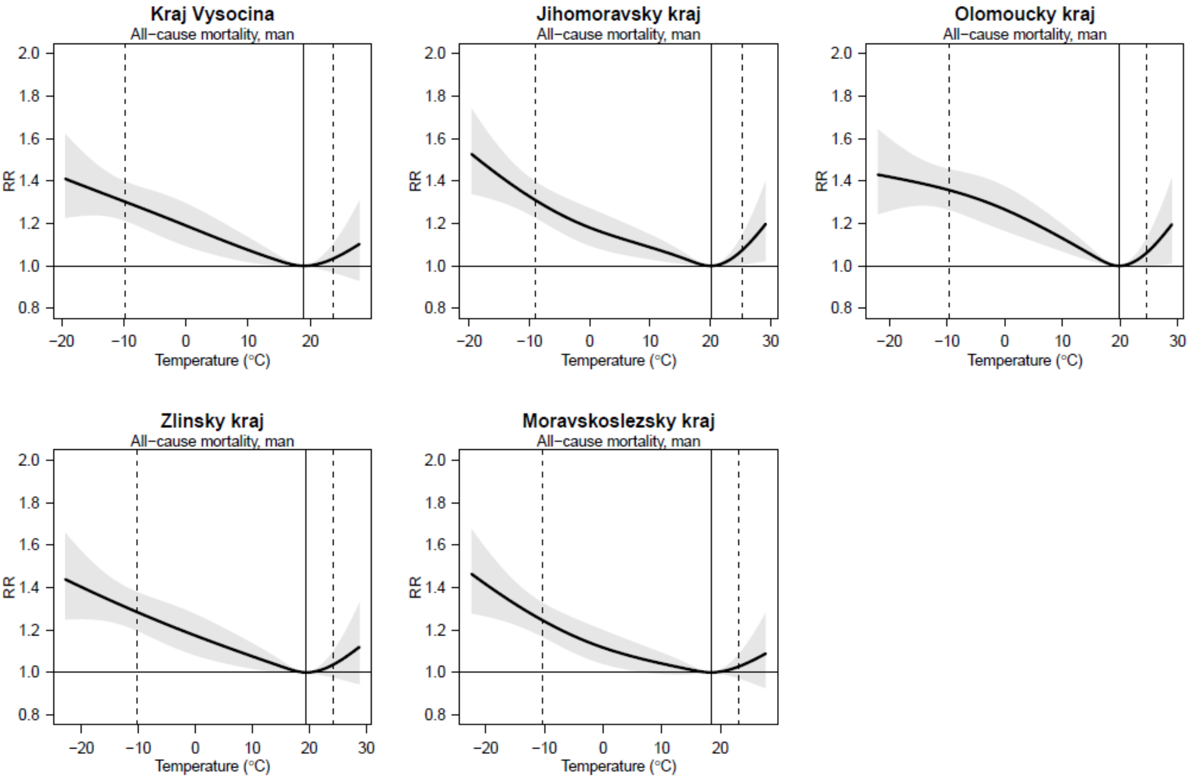
Kraj Vysocina
All−cause mortality, man
Jihomoravsky kraj
All−cause mortality, man
Olomoucky kraj
All−cause mortality, man
Zlinsky kraj
All−cause mortality, man
Moravskoslezsky kraj
All−cause mortality, man
RR
Temperature (°C)

Supplementary Figure S4 Temperature-mortality associations among women population in the regions of the Czech Republic from model for whole study period. RR=relative risk. Dashed vertical lines denote the 1st and 99th percentiles of the specific temperature distribution. Solid lines denote specific MMT.

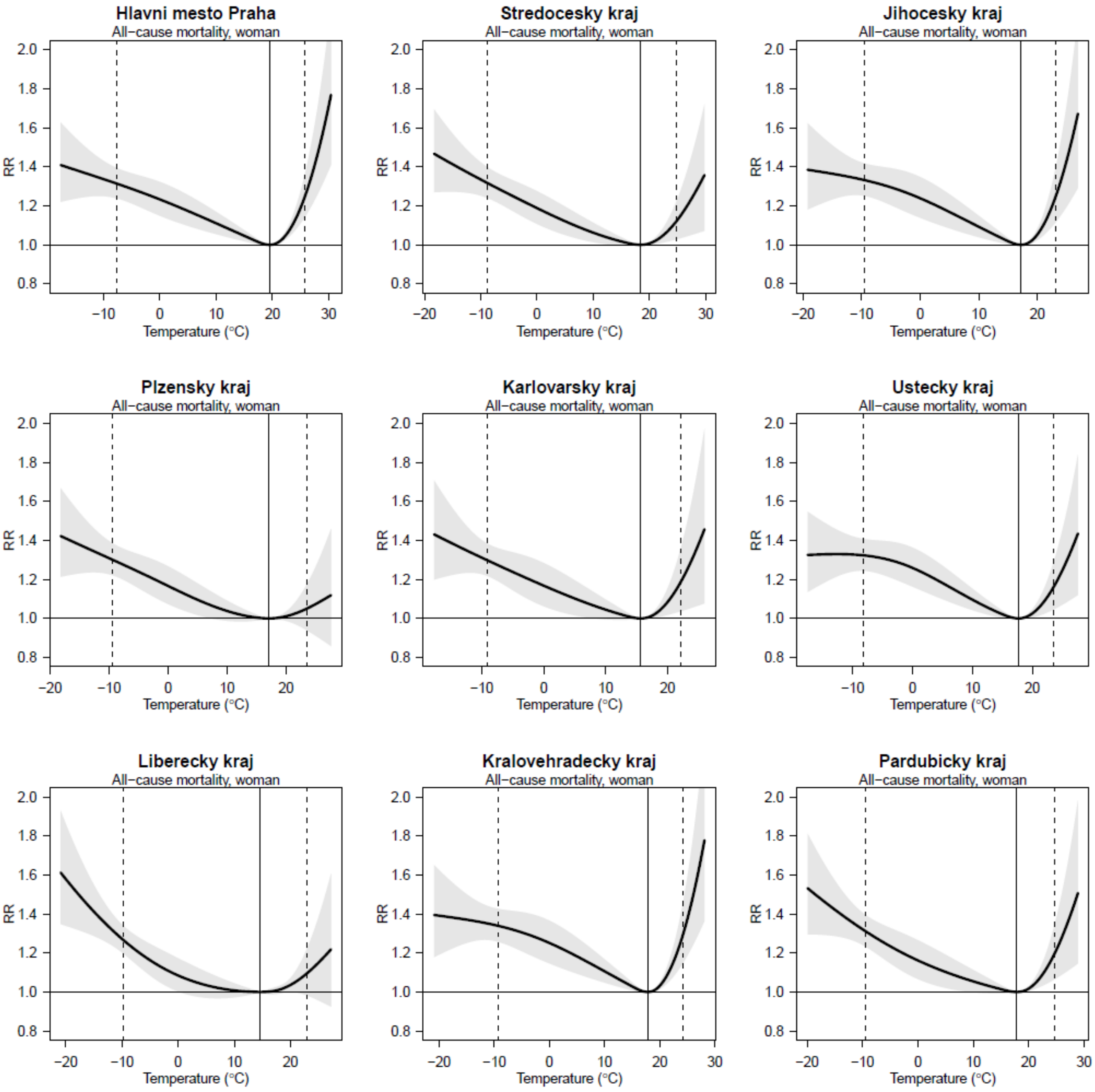

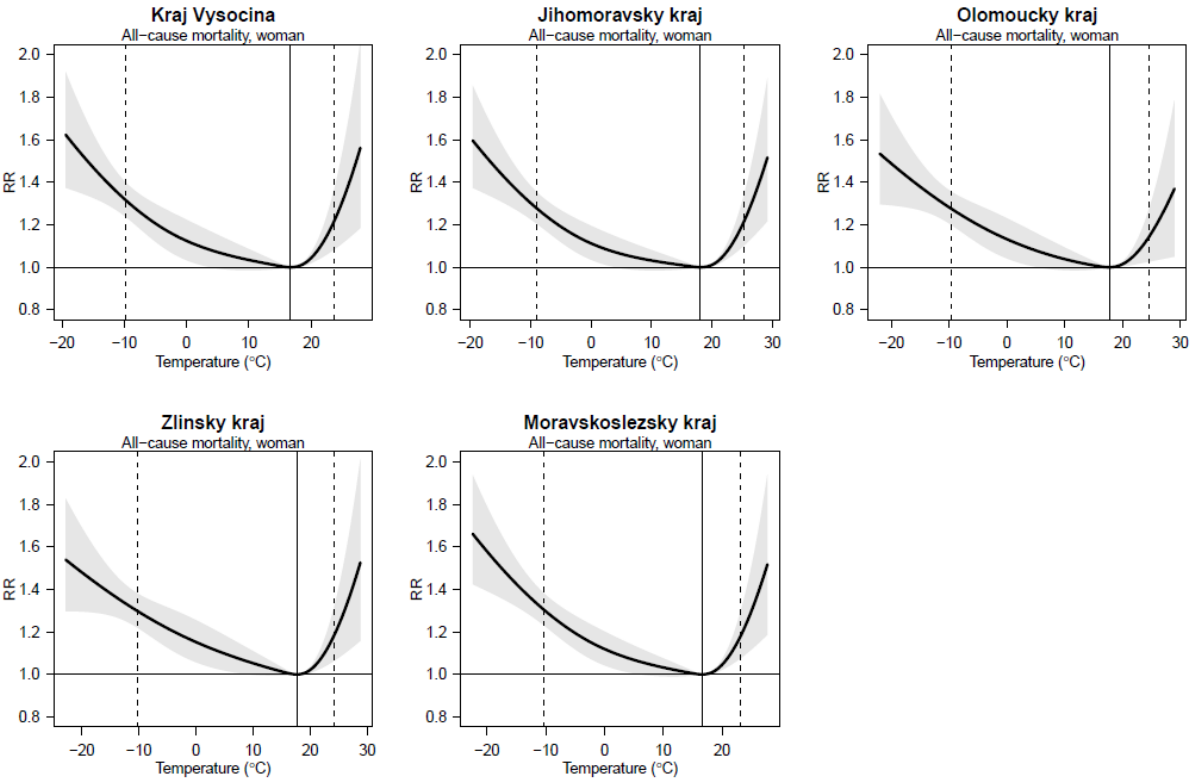
Kraj Vysocina
All−cause mortality, woman
Jihomoravsky kraj
All−cause mortality, woman
Olomoucky kraj
All−cause mortality, woman
Zlinsky kraj
All−cause mortality, woman
Moravskoslezsky kraj
All−cause mortality, woman
RR
Temperature (°C)
2.0
1.8
1.6
1.4
1.2
1.0
0.8
−20
−10
0
10
20
30

Supplementary Figure S5 Temperature-mortality associations among population aged 60-74 in the regions of the Czech Republic from model for whole study period. RR=relative risk. Dashed vertical lines denote the 1st and 99th percentiles of the specific temperature distribution. Solid lines denote specific MMT.

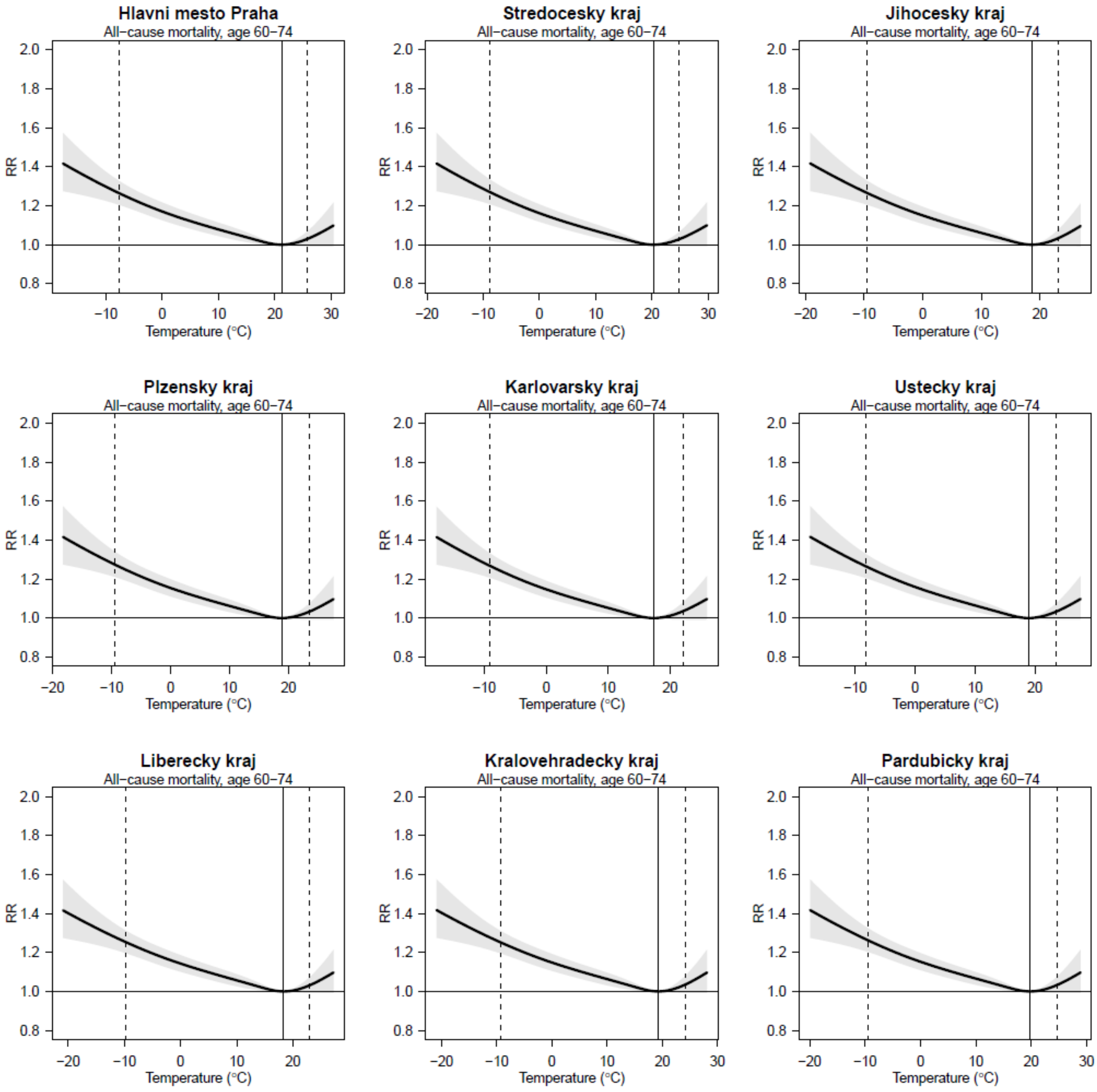

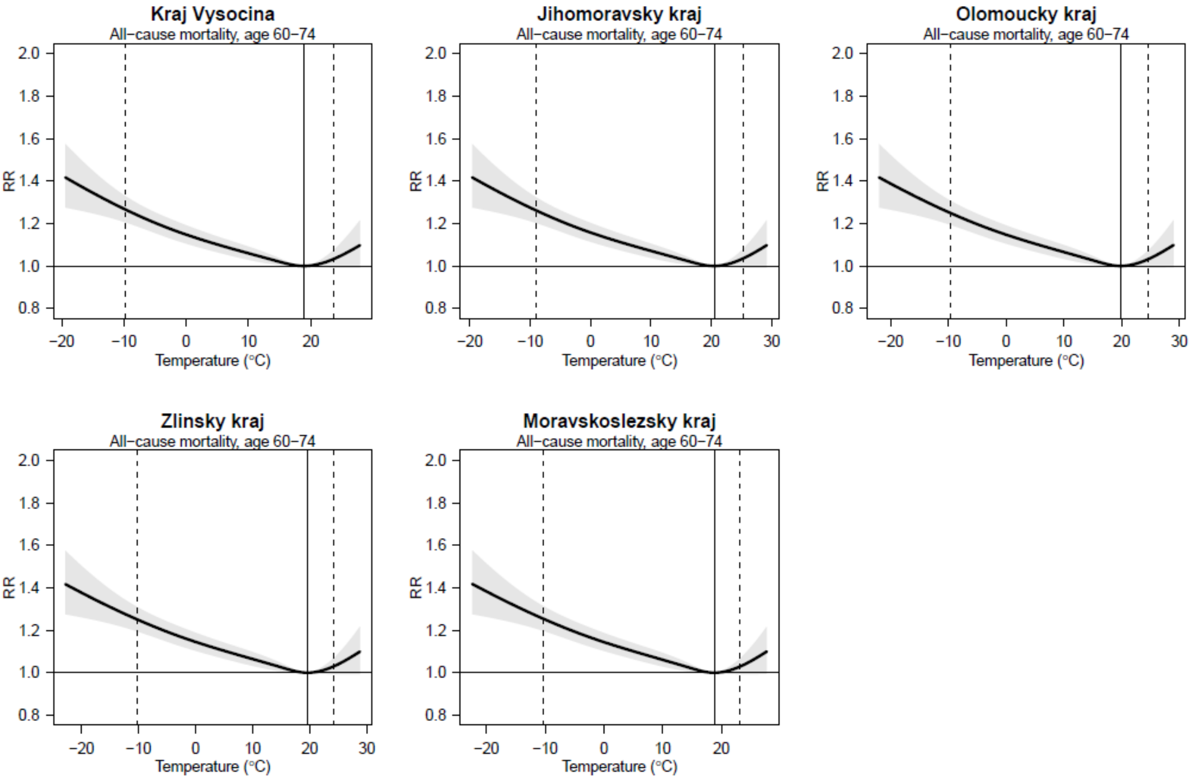

Kraj Vysocina
All−cause mortality, age 60−74
Jihomoravsky kraj
All−cause mortality, age 60−74
Olomoucky kraj
All−cause mortality, age 60−74
Zlinsky kraj
All−cause mortality, age 60−74
Moravskoslezsky kraj
All−cause mortality, age 60−74
RR
Temperature (°C)

Supplementary Figure S6 Temperature-mortality associations among population aged 75-89 in the regions of the Czech Republic from model for whole study period. RR=relative risk. Dashed vertical lines denote the 1st and 99th percentiles of the specific temperature distribution. Solid lines denote specific MMT.

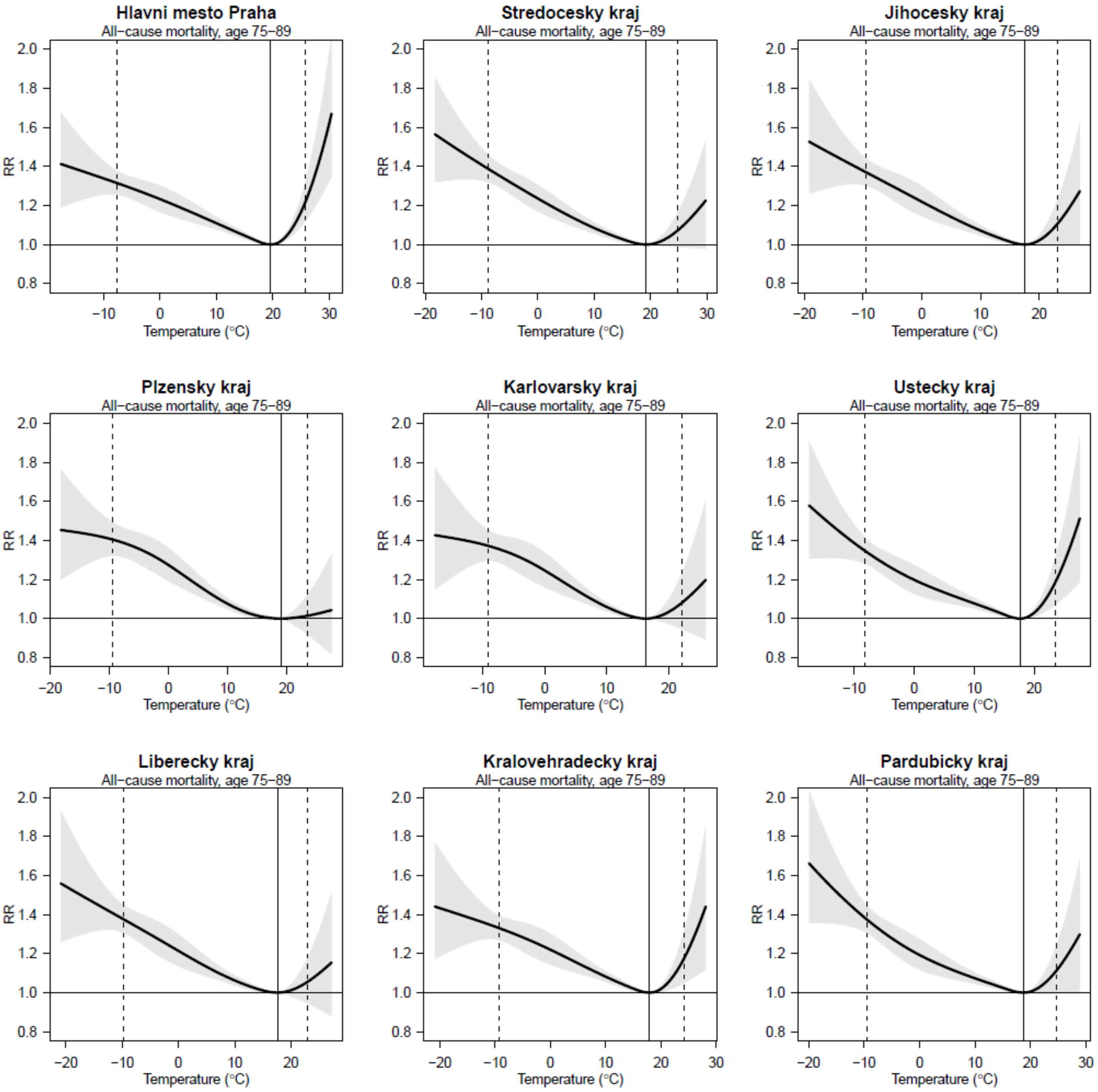

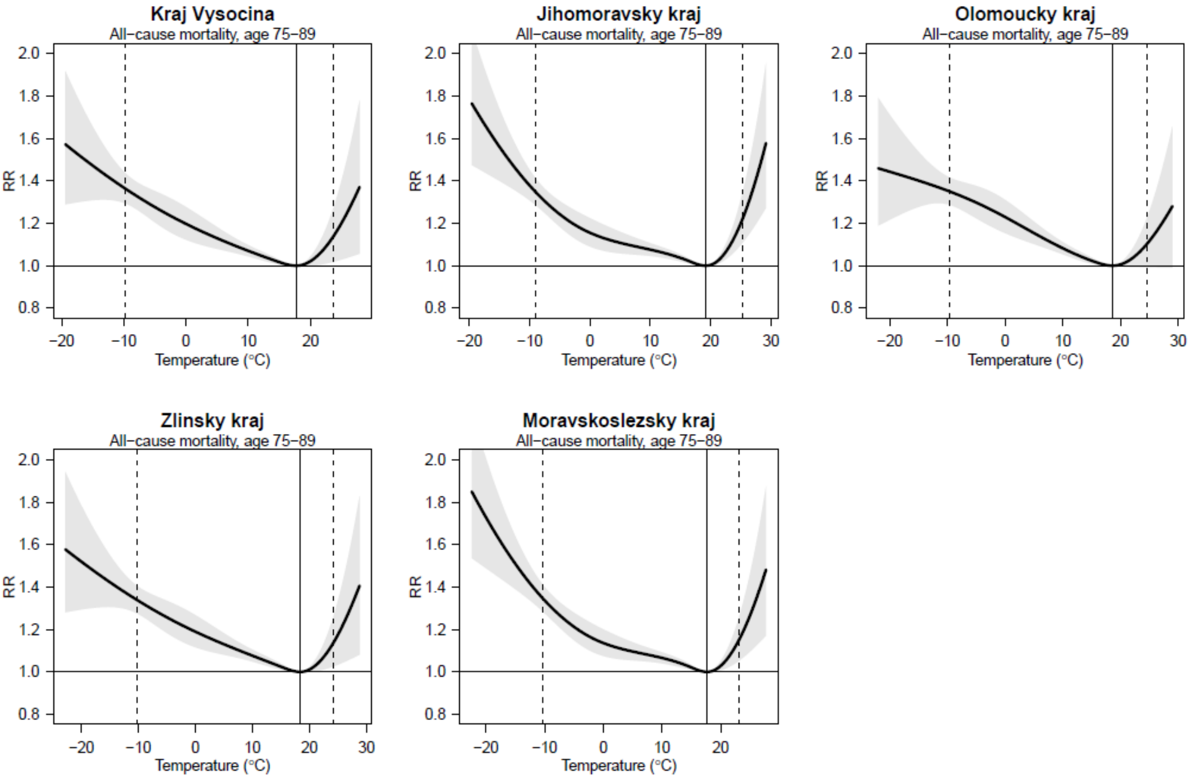
Kraj Vysocina
All−cause mortality, age 75−89
Jihomoravsky kraj
All−cause mortality, age 75−89
Olomoucky kraj
All−cause mortality, age 75−89
Zlinsky kraj
All−cause mortality, age 75−89
Moravskoslezsky kraj
All−cause mortality, age 75−89
RR
Temperature (°C)
2.0
1.8
1.6
1.4
1.2
1.0
0.8
−20
−10
0
10
20
30

Supplementary Figure S7 Temperature-mortality associations among population aged 90+ in the regions of the Czech Republic from model for whole study period. RR=relative risk. Dashed vertical lines denote the 1st and 99th percentiles of the specific temperature distribution. Solid lines denote specific MMT.

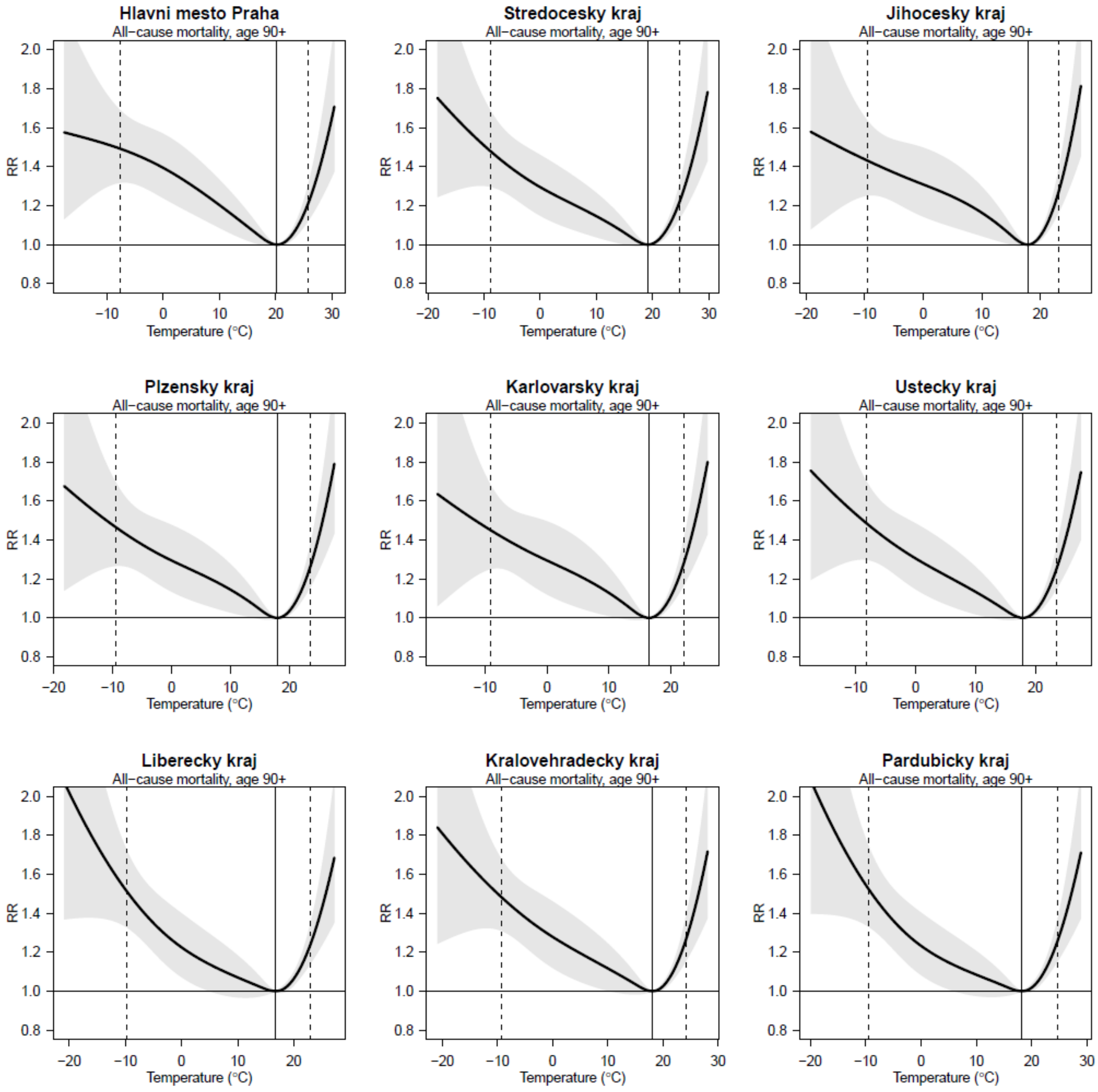

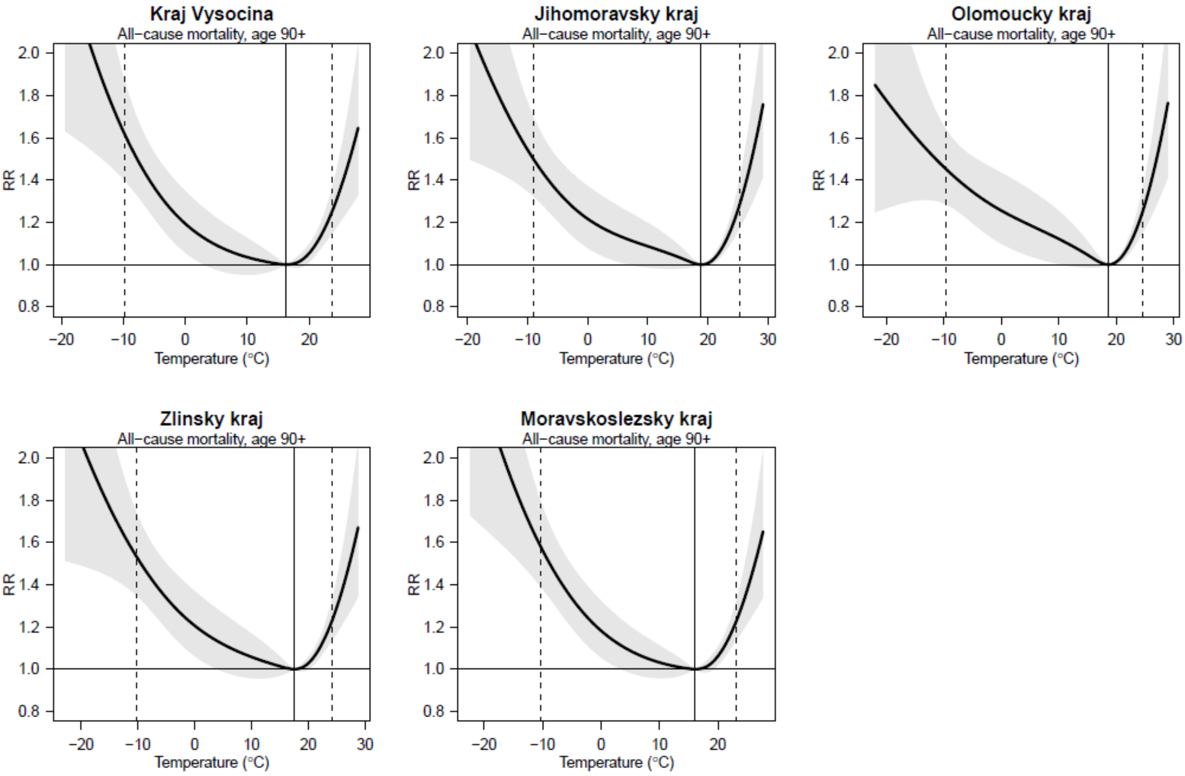
Kraj Vysocina
All−cause mortality, age 90+
Jihomoravsky kraj
All−cause mortality, age 90+
Olomoucky kraj
All−cause mortality, age 90+
Zlinsky kraj
All−cause mortality, age 90+
Moravskoslezsky kraj
All−cause mortality, age 90+
RR
Temperature (°C)

Supplementary Figure S8 Temperature-mortality associations from cardiovascular diseases among overall population in the regions of the Czech Republic from model for whole study period. RR=relative risk. Dashed vertical lines denote the 1st and 99th percentiles of the specific temperature distribution. Solid lines denote specific MMT.

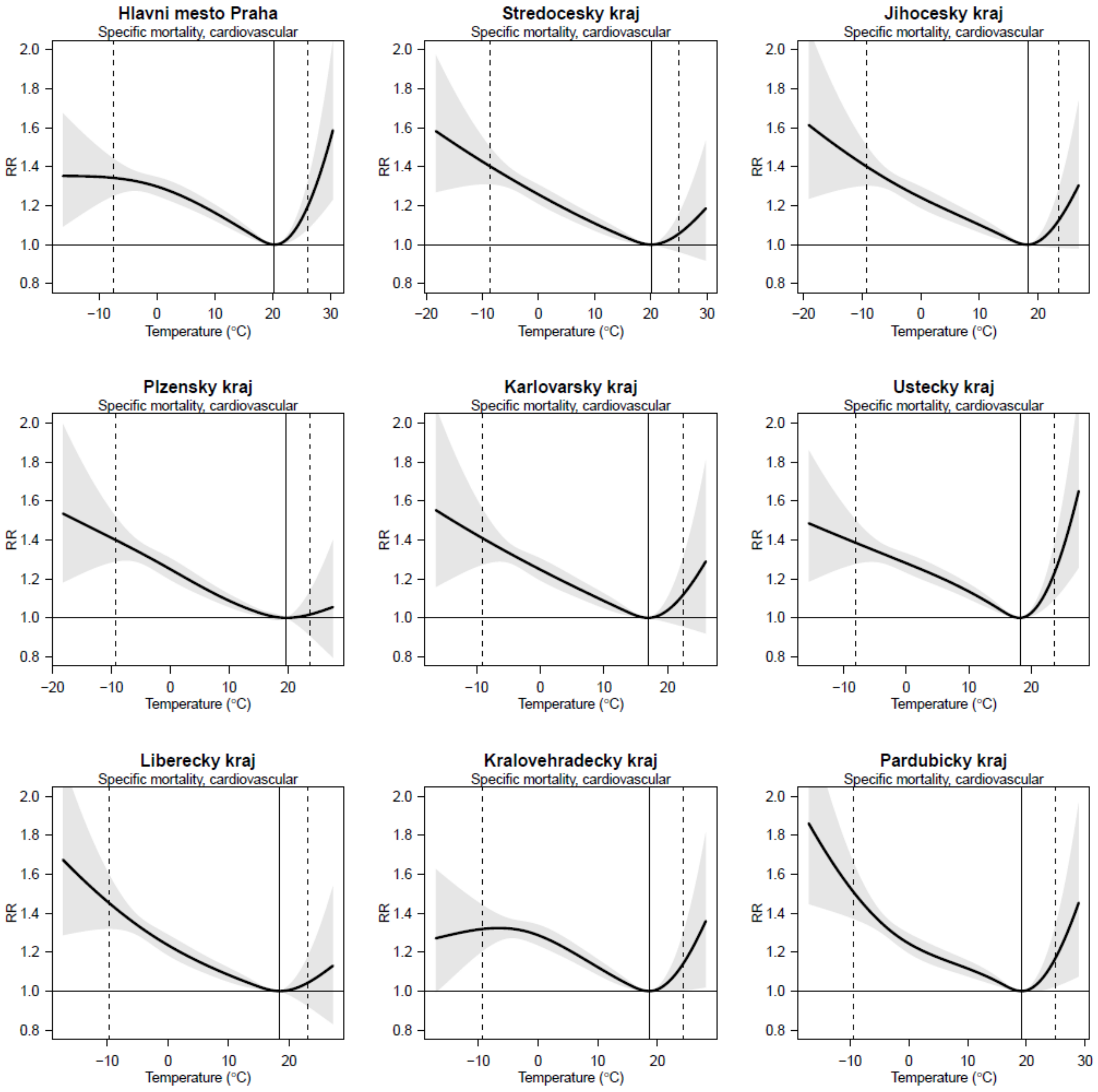

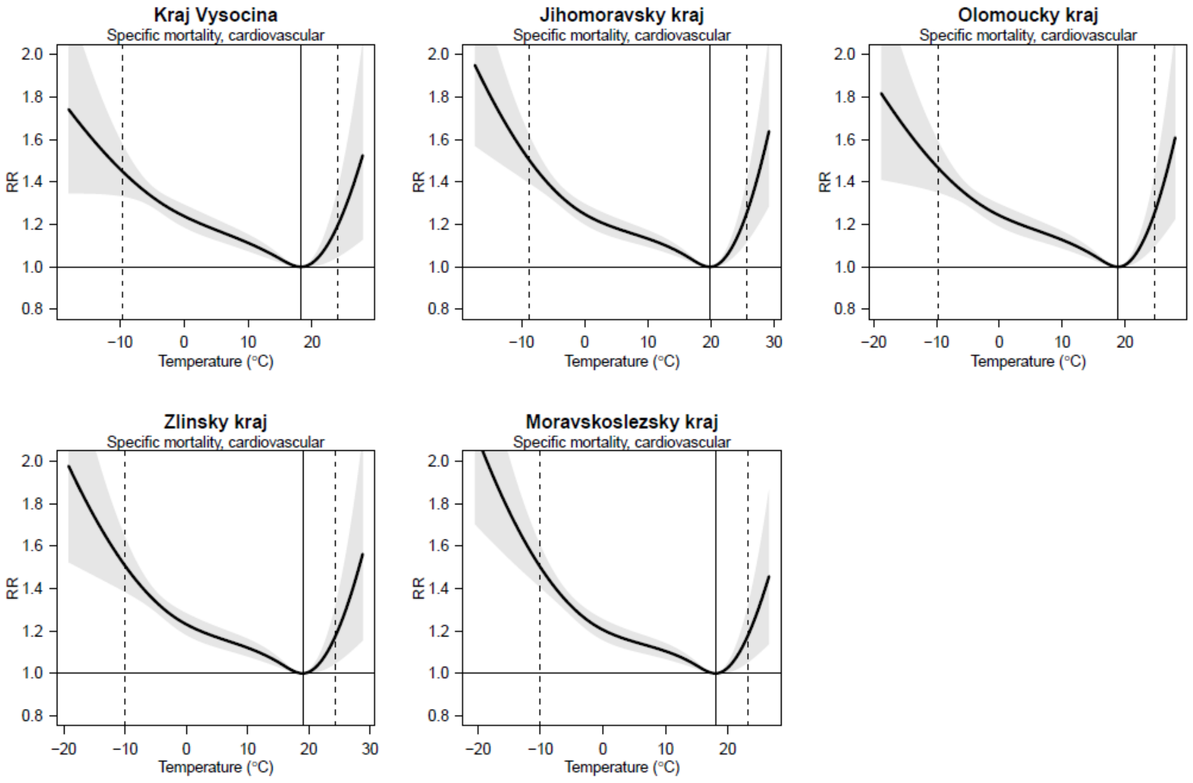
Kraj Vysocina
Specific mortality, cardiovascular
Jihomoravsky kraj
Specific mortality, cardiovascular
Olomoucky kraj
Specific mortality, cardiovascular
Zlinsky kraj
Specific mortality, cardiovascular
Moravskoslezsky kraj
Specific mortality, cardiovascular
RR
0.8
1.0
1.2
1.4
1.6
1.8
2.0
Temperature (°C)
−20
−10
0
10
20
30

Supplementary Figure S9 Temperature-mortality associations from respiratory diseases among overall population in the regions of the Czech Republic from model for whole study period. RR=relative risk. Dashed vertical lines denote the 1st and 99th percentiles of the specific temperature distribution. Solid lines denote specific MMT.

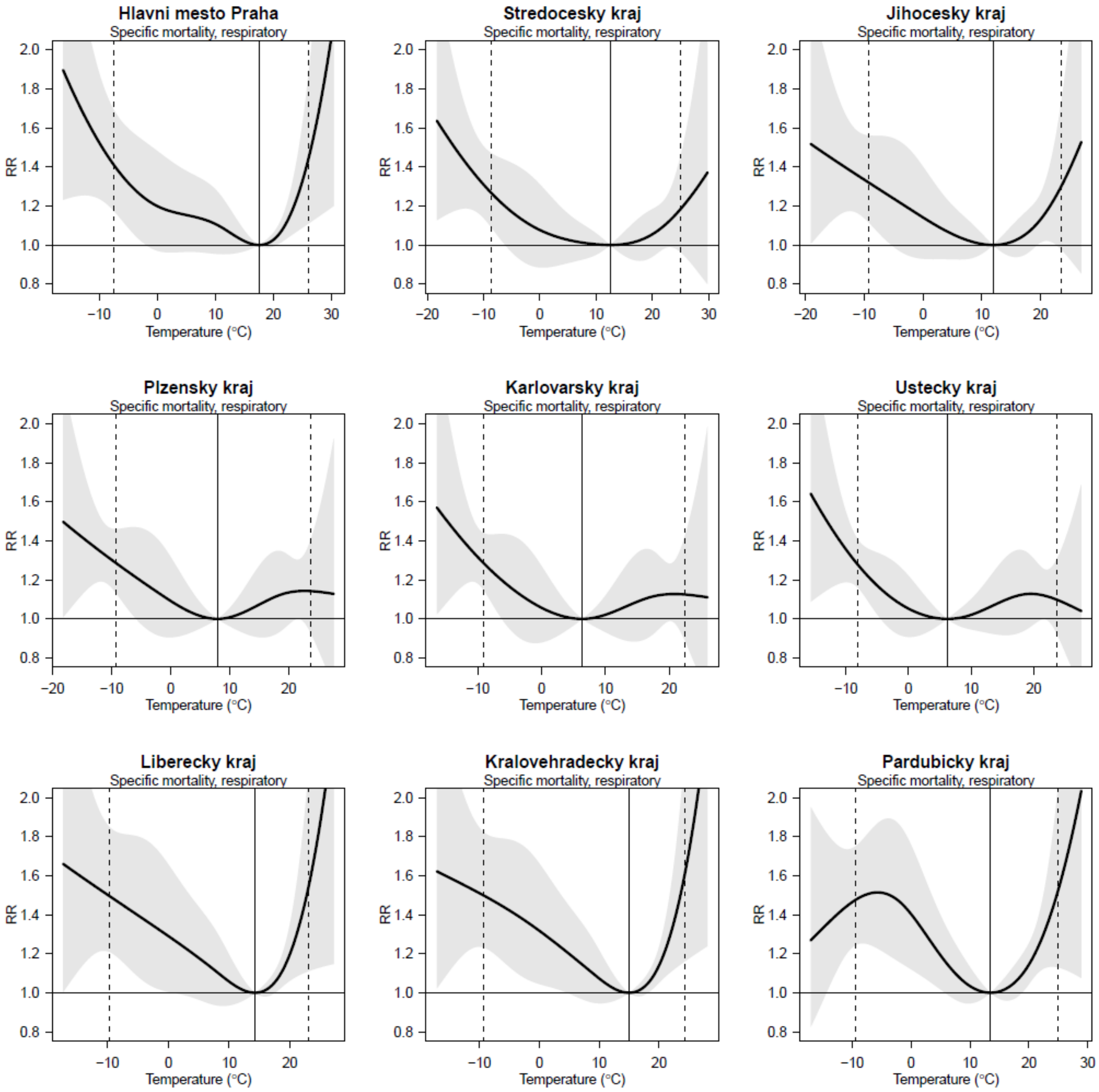

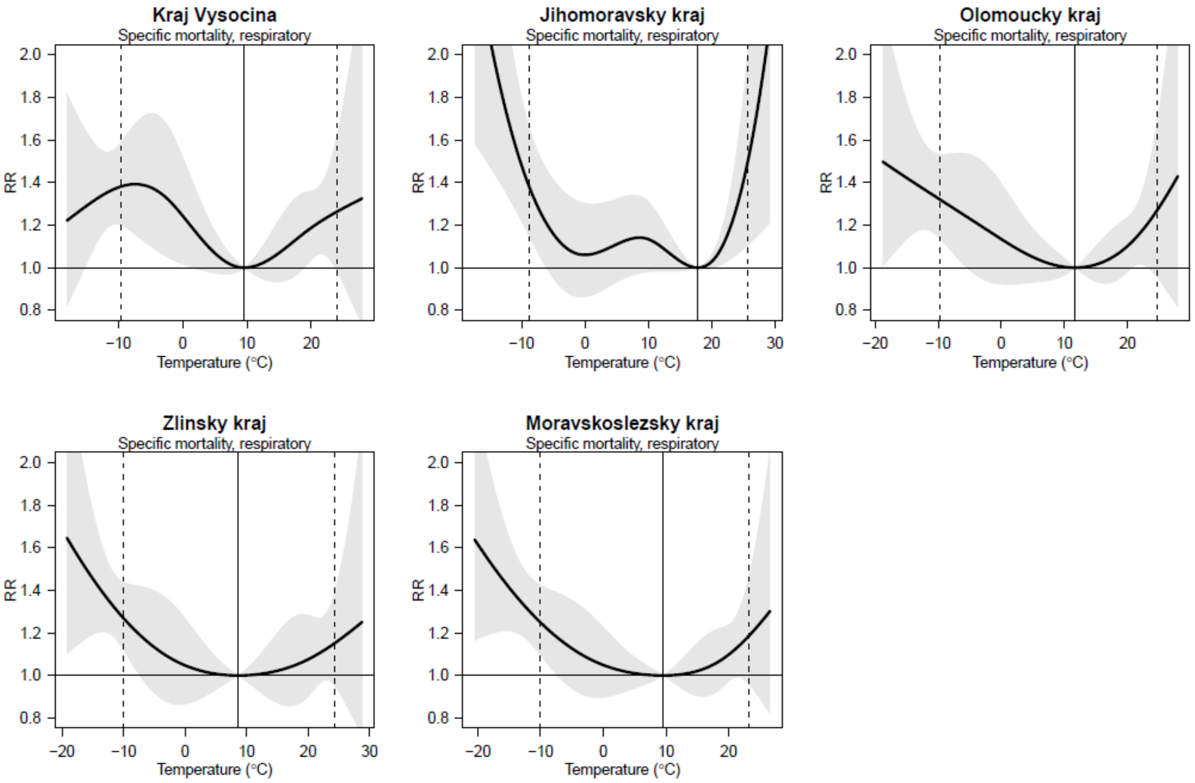
Kraj Vysocina
Specific mortality, respiratory
Jihomoravsky kraj
Specific mortality, respiratory
Olomoucky kraj
Specific mortality, respiratory
Zlinsky kraj
Specific mortality, respiratory
Moravskoslezsky kraj
Specific mortality, respiratory
RR
Temperature (°C)

Supplementary Figure S10 Trend in RR of death at 1st (cold) and 99th (heat) temperature percentile among overall population in the regions of the Czech Republic from model with subsets of 13-year moving periods. RR=relative risk, TP01=temperature percentile 1, TP99=temperature percentile 99.

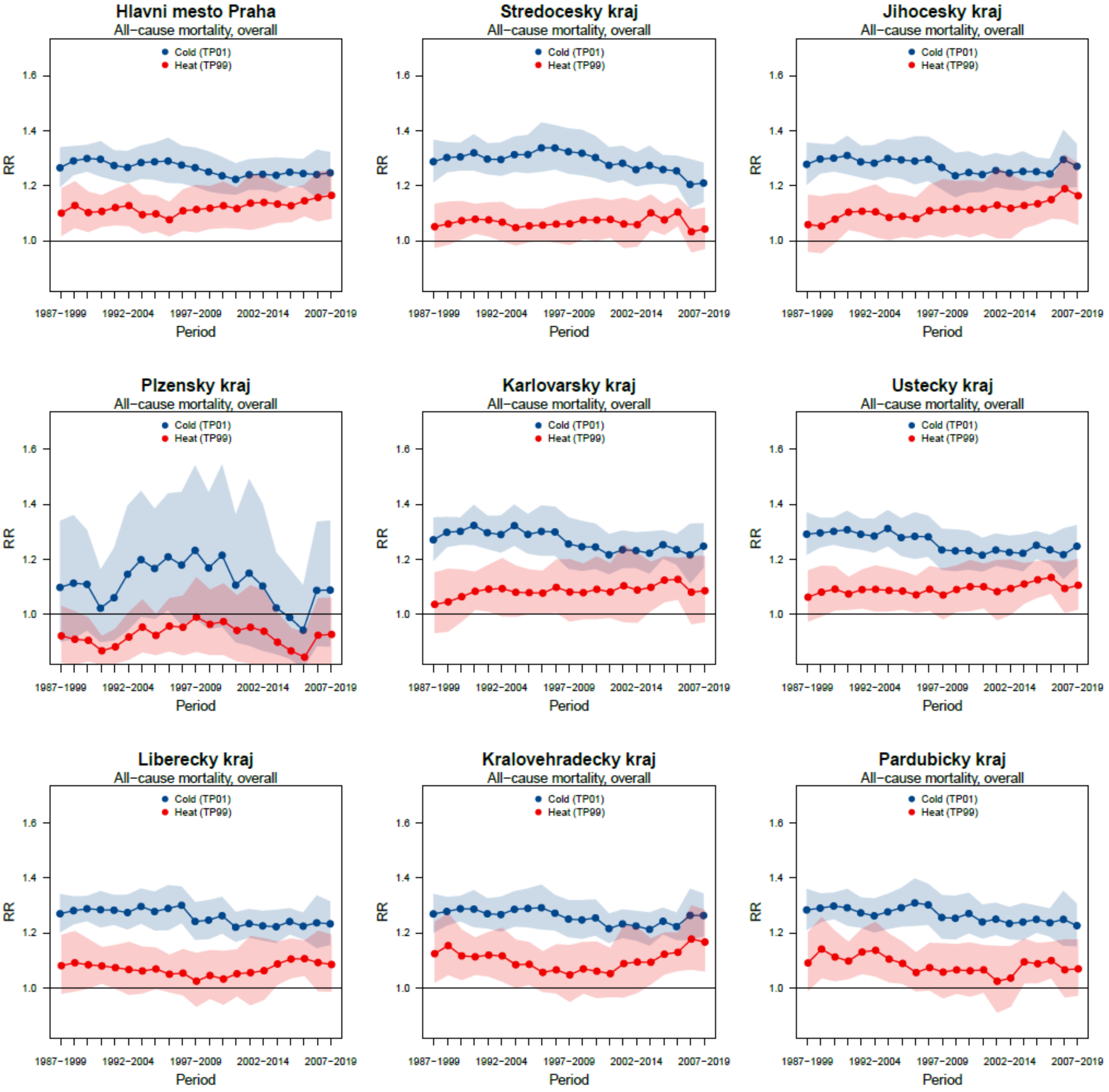

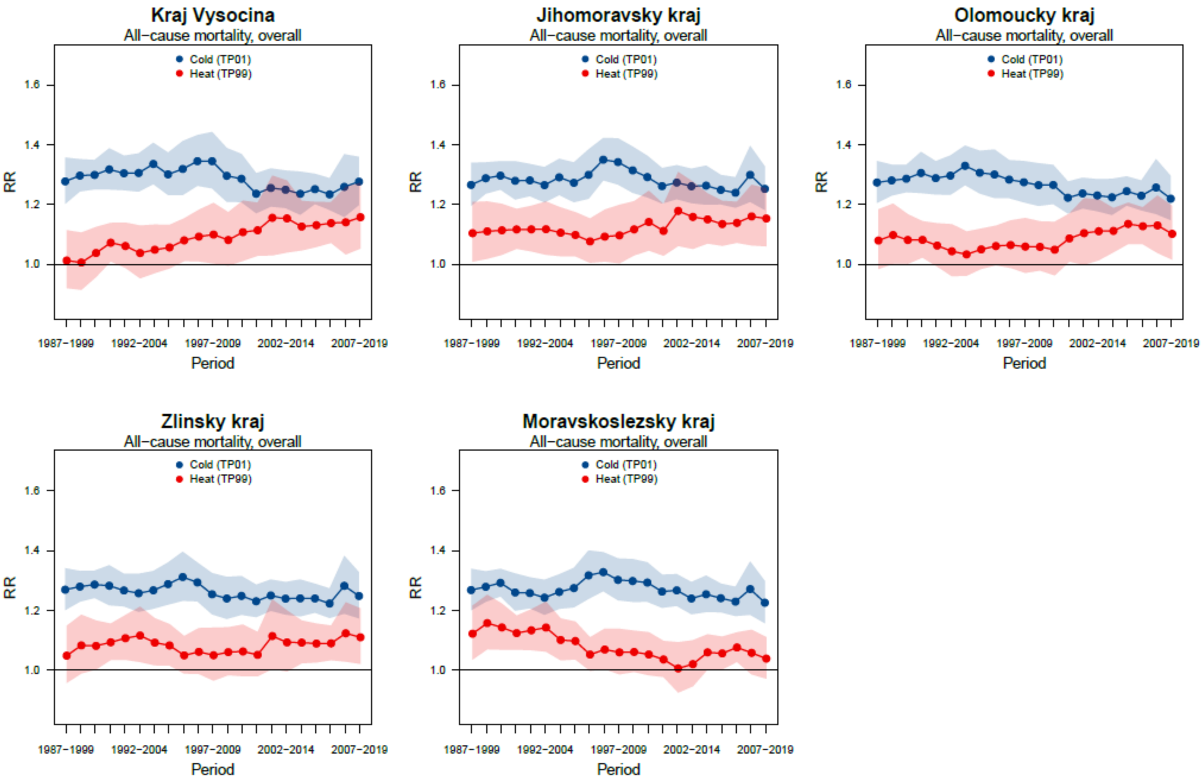
Kraj Vysocina
All−cause mortality, overall
Cold (TP01)
Heat (TP99)
RR
1.0
1.2
1.4
1.6
1987−1999
1992−2004
1997−2009
2002−2014
2007−2019
Period
Jihomoravsky kraj
All−cause mortality, overall
Cold (TP01)
Heat (TP99)
RR
Period
Olomoucky kraj
All−cause mortality, overall
Cold (TP01)
Heat (TP99)
RR
Period
Zlinsky kraj
All−cause mortality, overall
Cold (TP01)
Heat (TP99)
RR
Period
Moravskoslezsky kraj
All−cause mortality, overall
Cold (TP01)
Heat (TP99)
RR
Period

Supplementary Figure S11 Trend in RR at 1st (cold) and 99th (heat) temperature percentile among men population in the regions of the Czech Republic from model with subsets of 13-year moving periods. RR=relative risk, TP01=temperature percentile 1, TP99=temperature percentile 99.

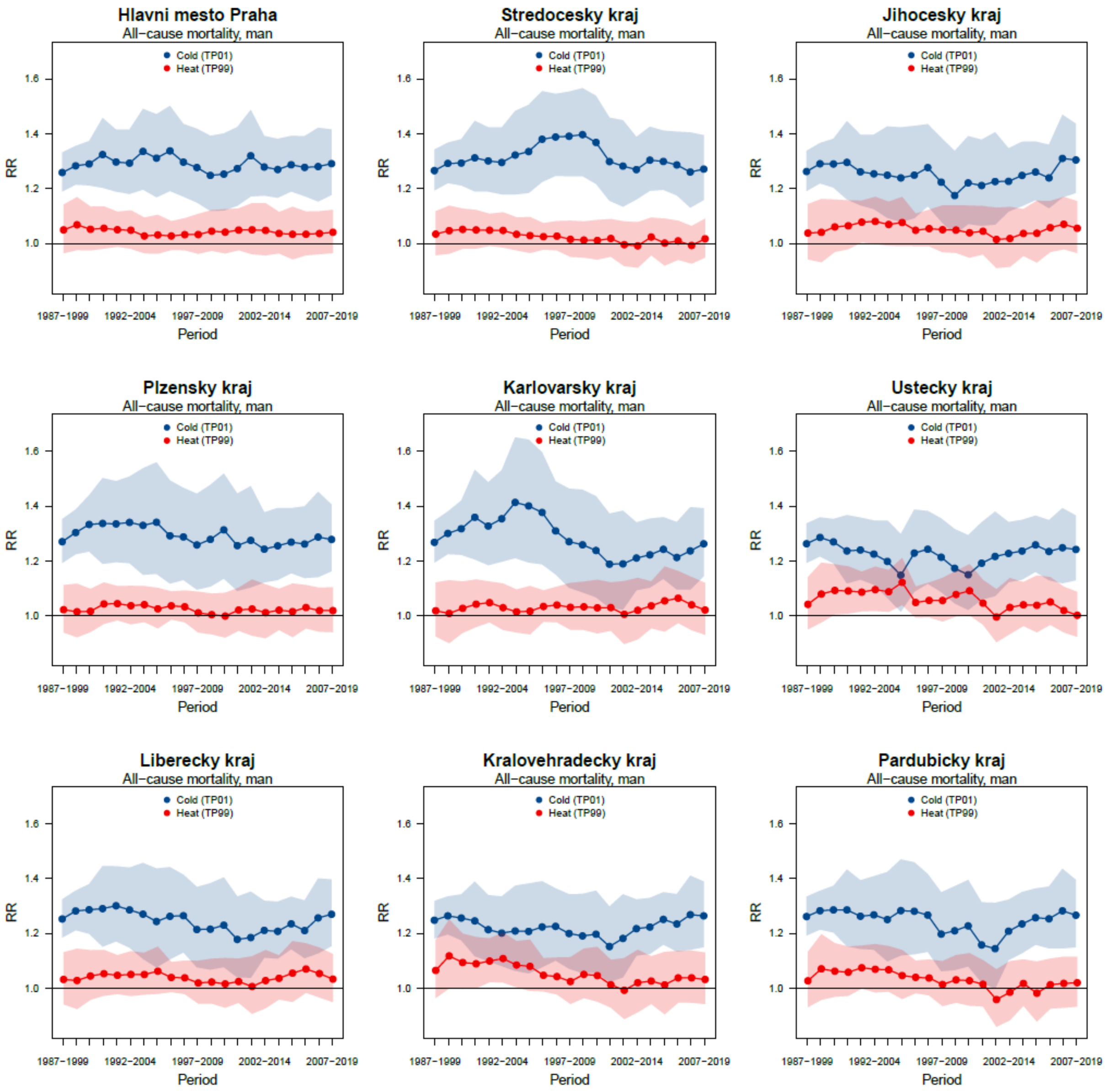

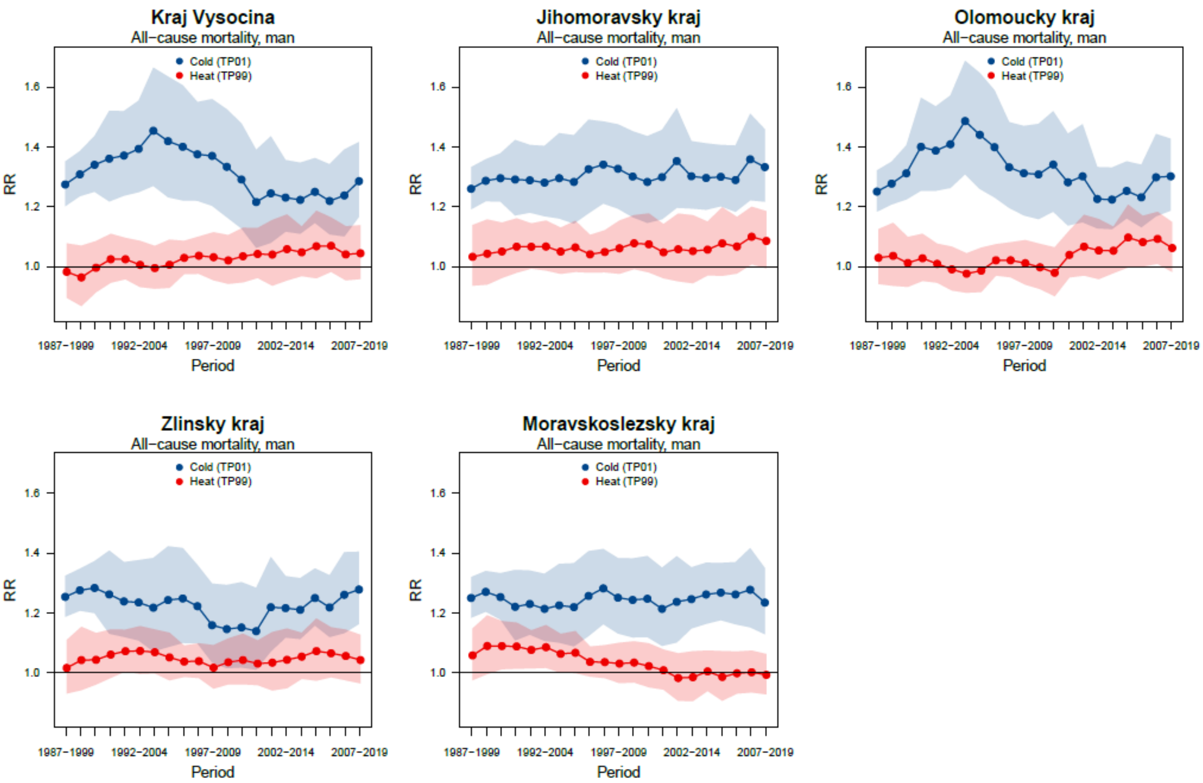
Kraj Vysocina
All−cause mortality, man
Jihomoravsky kraj
All−cause mortality, man
Olomoucky kraj
All−cause mortality, man
Zlinsky kraj
All−cause mortality, man
Moravskoslezsky kraj
All−cause mortality, man
Cold (TP01)
Heat (TP99)
RR
1.0
1.2
1.4
1.6
1987−1999
1992−2004
1997−2009
2002−2014
2007−2019
Period

Supplementary Figure S12 Trend in RR of death at 1st (cold) and 99th (heat) temperature percentile among women population in the regions of the Czech Republic from model with subsets of 13-year moving periods. RR=relative risk, TP01=temperature percentile 1, TP99=temperature percentile 99.

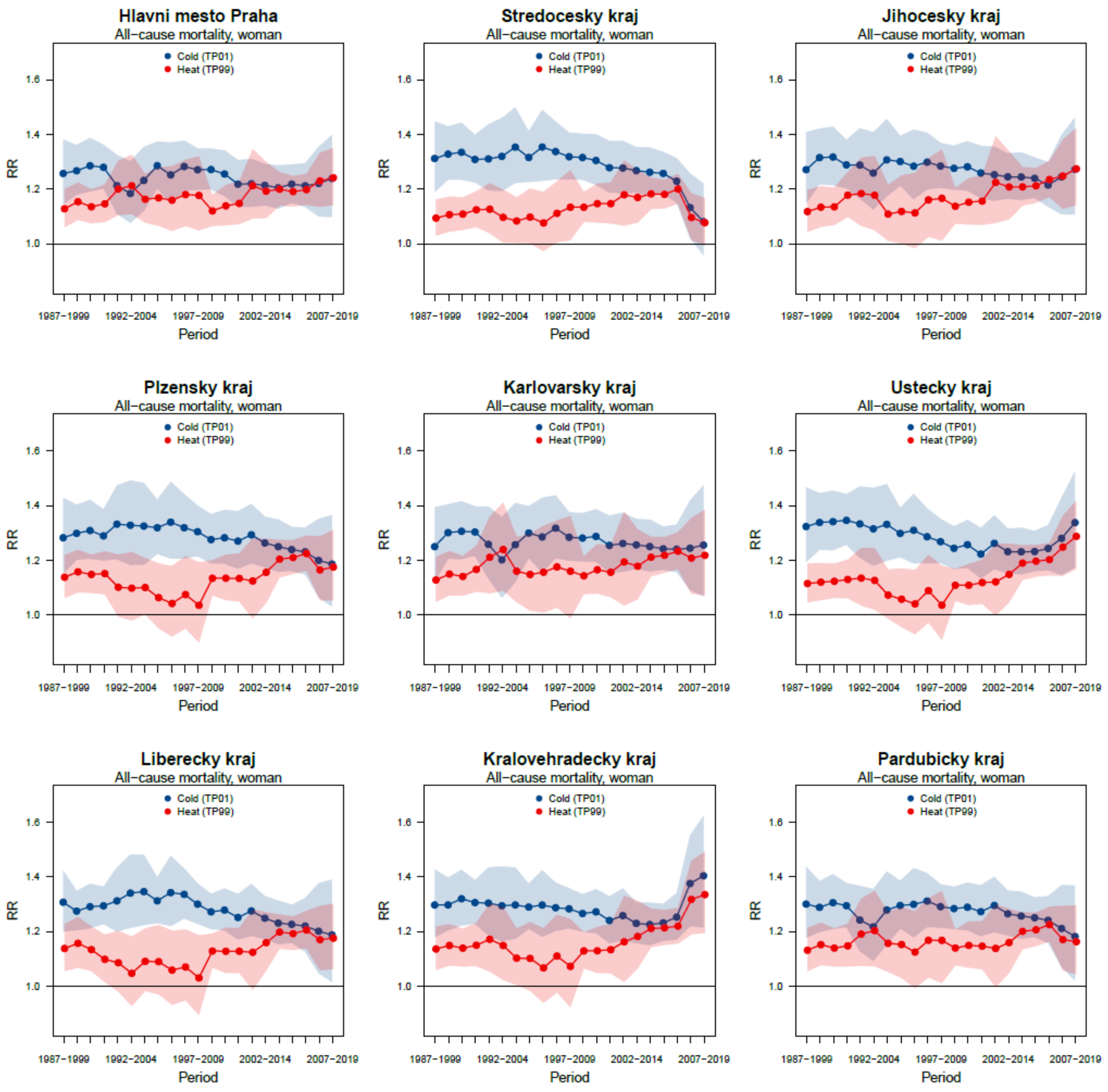

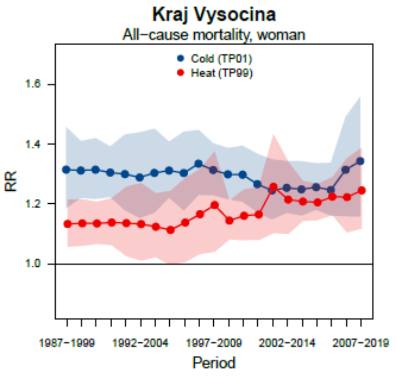
Kraj Vysocina
All−cause mortality, woman
Cold (TP01)
Heat (TP99)
RR
1.0
1.2
1.4
1.6
1987−1999
1992−2004
1997−2009
2002−2014
2007−2019
Period

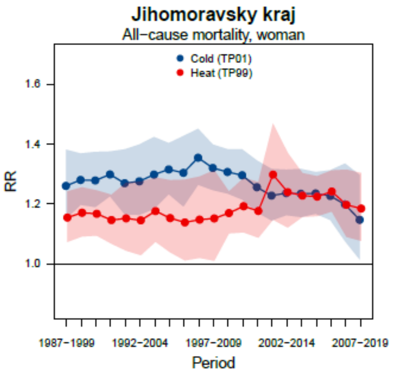
Jihomoravsky kraj
All−cause mortality, woman
Cold (TP01)
Heat (TP99)
RR
1.0
1.2
1.4
1.6
1987−1999
1992−2004
1997−2009
2002−2014
2007−2019
Period

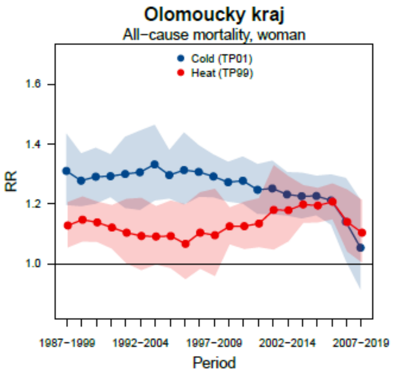
Olomoucky kraj
All−cause mortality, woman
Cold (TP01)
Heat (TP99)
RR
1.0
1.2
1.4
1.6
1987−1999
1992−2004
1997−2009
2002−2014
2007−2019
Period

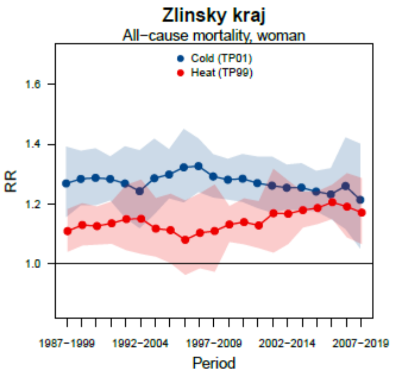
Zlinsky kraj
All−cause mortality, woman
Cold (TP01)
Heat (TP99)
RR
1.0
1.2
1.4
1.6
1987−1999
1992−2004
1997−2009
2002−2014
2007−2019
Period

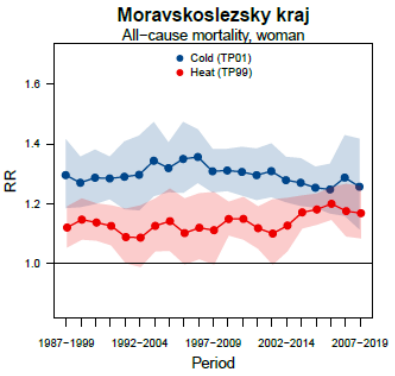
Moravskoslezsky kraj
All−cause mortality, woman
Cold (TP01)
Heat (TP99)
RR
1.0
1.2
1.4
1.6
1987−1999
1992−2004
1997−2009
2002−2014
2007−2019
Period

Supplementary Figure S13 Trend in RR of death at 1st (cold) and 99th (heat) temperature percentile among population aged 60-74 in the regions of the Czech Republic from model with subsets of 13-year moving periods. RR=relative risk, TP01=temperature percentile 1, TP99=temperature percentile 99.

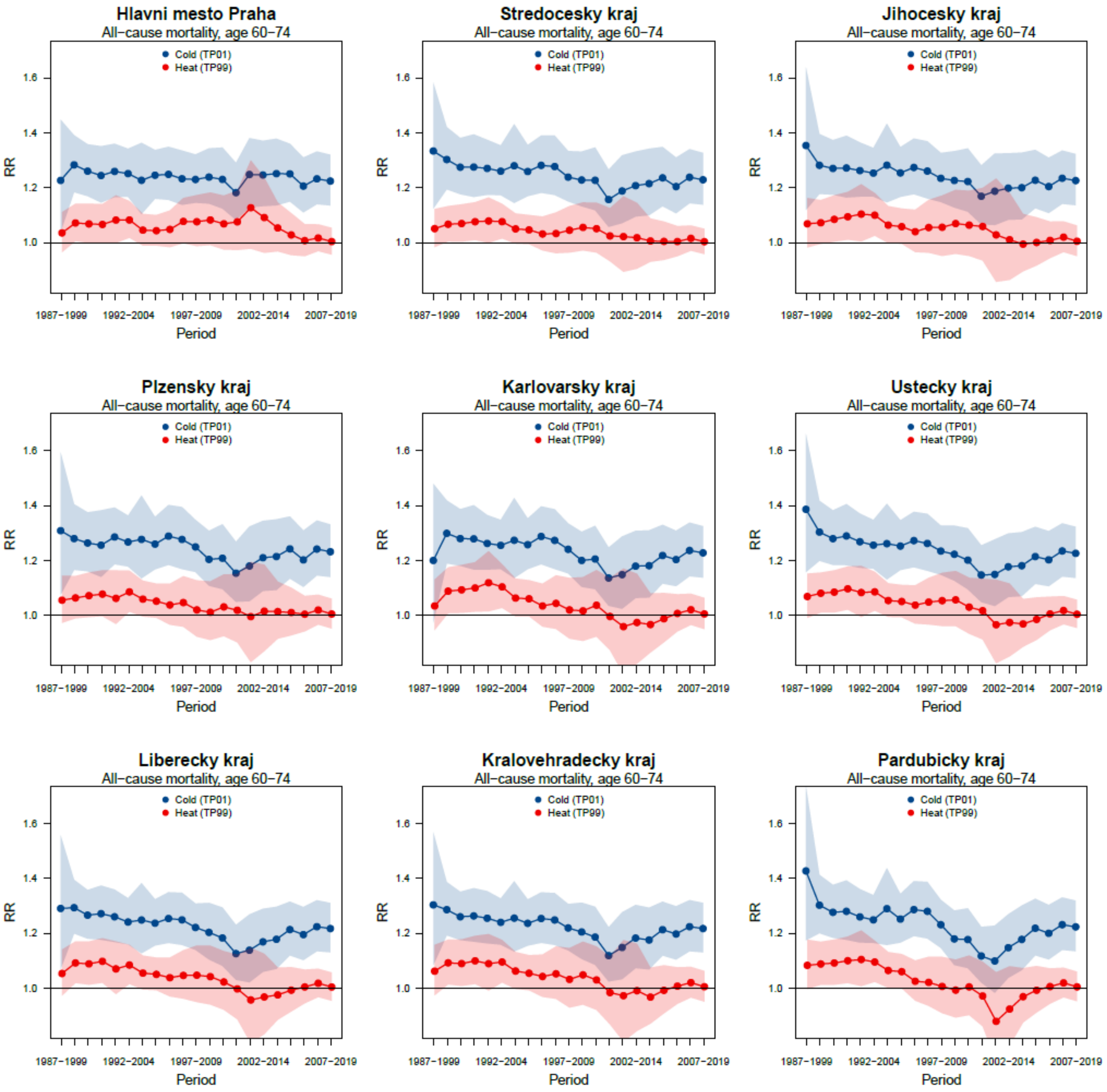

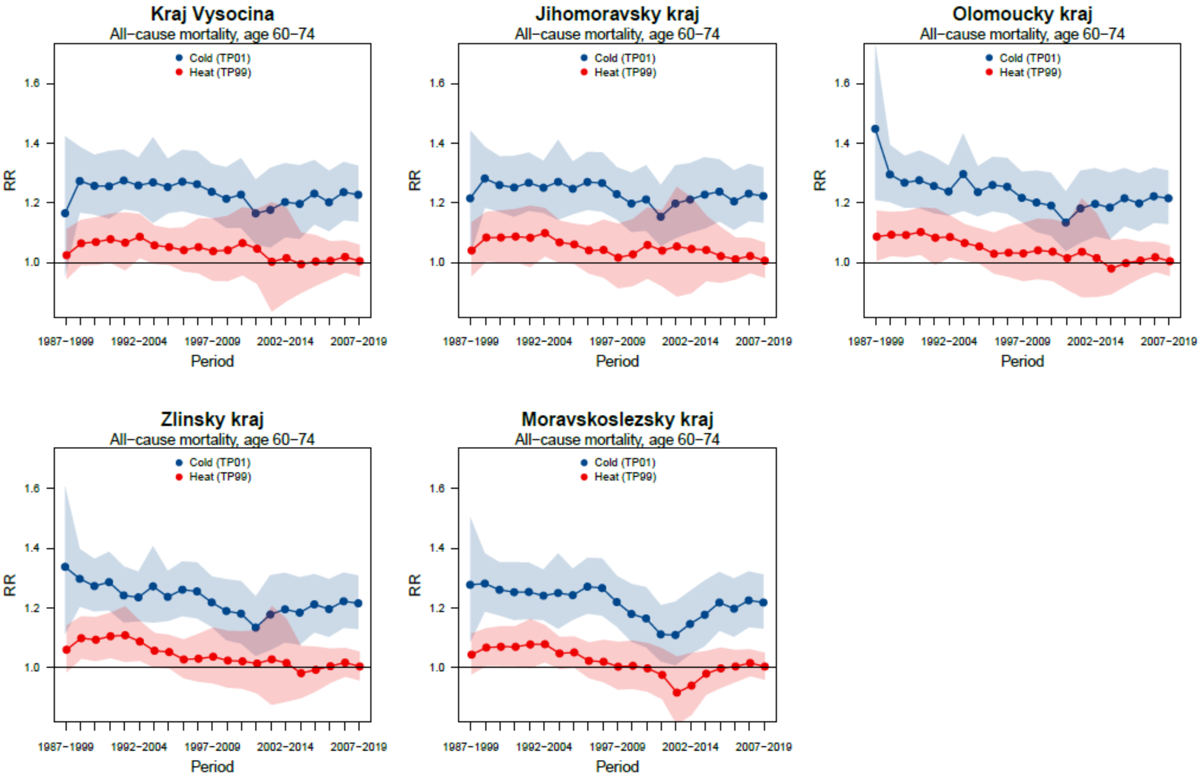
Kraj Vysocina
All−cause mortality, age 60−74
Cold (TP01)
Heat (TP99)
RR
1.0
1.2
1.4
1.6
1987−1999
1992−2004
1997−2009
2002−2014
2007−2019
Period
Jihomoravsky kraj
All−cause mortality, age 60−74
Cold (TP01)
Heat (TP99)
RR
Period
Olomoucky kraj
All−cause mortality, age 60−74
Cold (TP01)
Heat (TP99)
RR
Period
Zlinsky kraj
All−cause mortality, age 60−74
Cold (TP01)
Heat (TP99)
RR
Period
Moravskoslezsky kraj
All−cause mortality, age 60−74
Cold (TP01)
Heat (TP99)
RR
Period

Supplementary Figure S14 Trend in RR of death at 1st (cold) and 99th (heat) temperature percentile among population aged 75-89 population in the regions of the Czech Republic from model with subsets of 13-year moving periods. RR=relative risk, TP01=temperature percentile 1, TP99=temperature percentile 99.

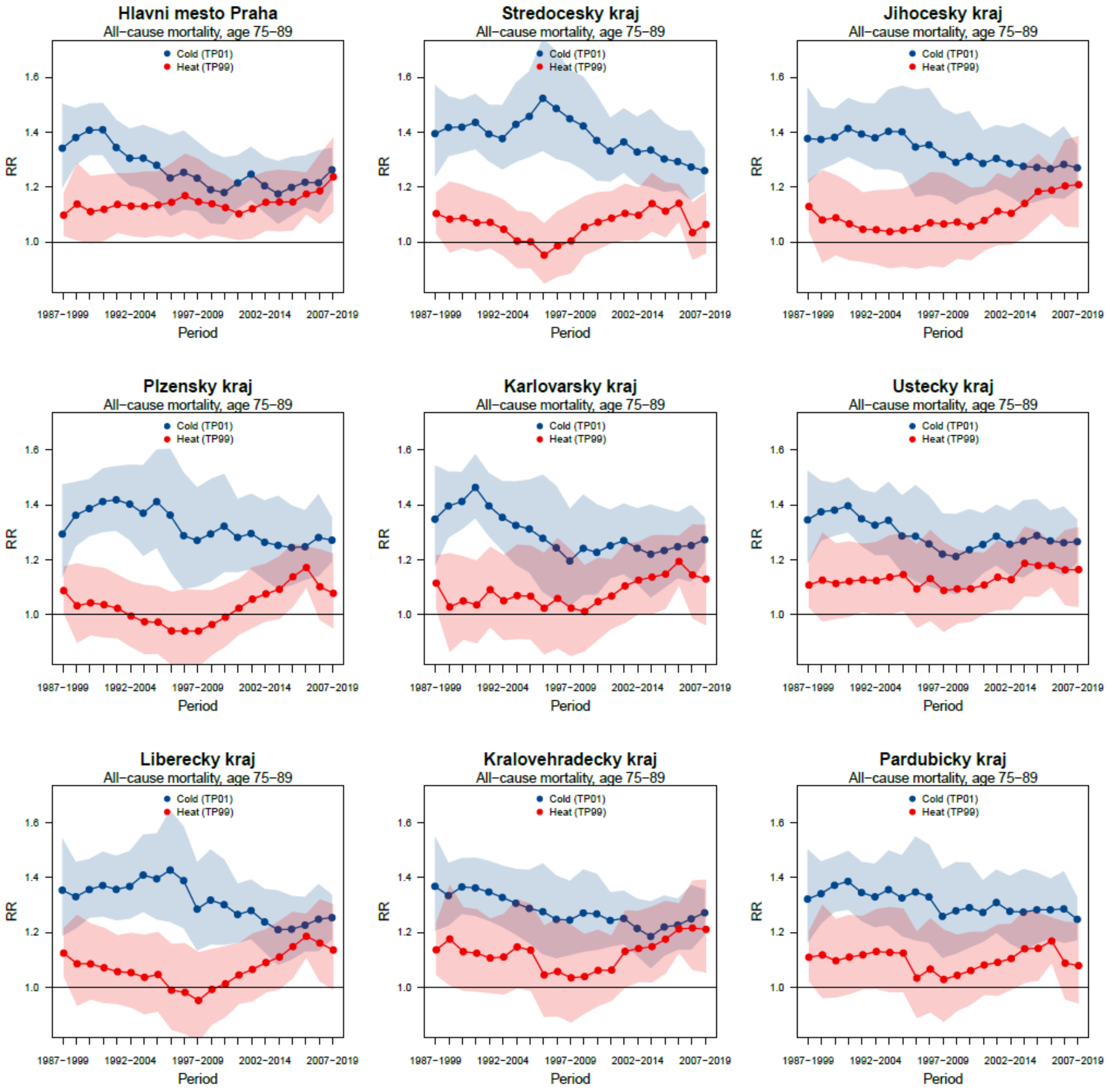

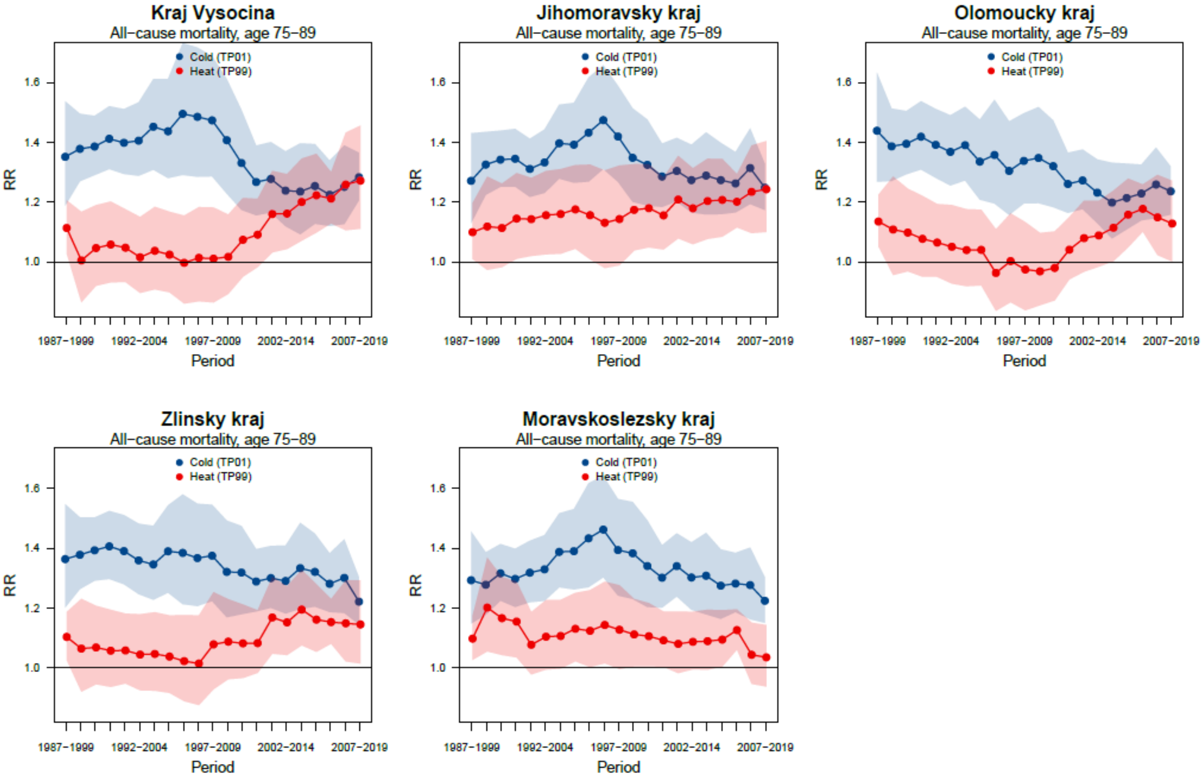
Kraj Vysocina
All−cause mortality, age 75−89
Cold (TP01)
Heat (TP99)
RR
1.0
1.2
1.4
1.6
1987−1999
1992−2004
1997−2009
2002−2014
2007−2019
Period
Jihomoravsky kraj
All−cause mortality, age 75−89
Olomoucky kraj
All−cause mortality, age 75−89
Zlinsky kraj
All−cause mortality, age 75−89
Moravskoslezsky kraj
All−cause mortality, age 75−89

Supplementary Figure S15 Trend in RR of death at 1st (cold) and 99th (heat) temperature percentile among population aged 90+ in the regions of the Czech Republic from model with subsets of 13-year moving periods. RR=relative risk, TP01=temperature percentile 1, TP99=temperature percentile 99.

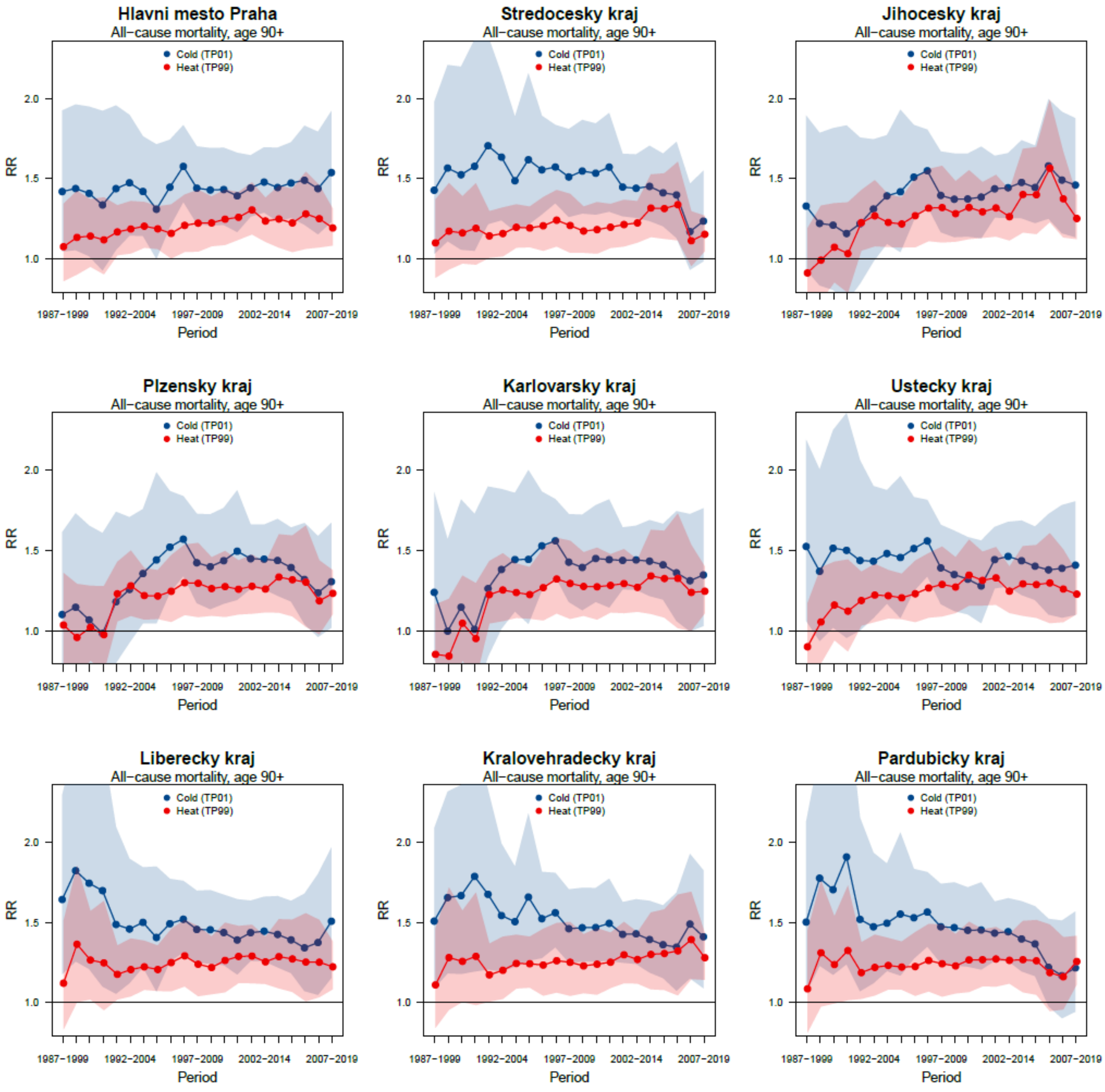

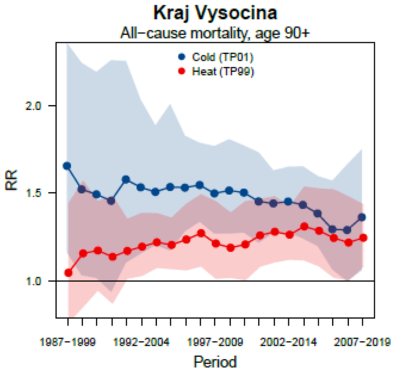
Kraj Vysocina
All−cause mortality, age 90+
Cold (TP01)
Heat (TP99)
RR
2.0
1.5
1.0
1987−1999
1992−2004
1997−2009
2002−2014
2007−2019
Period

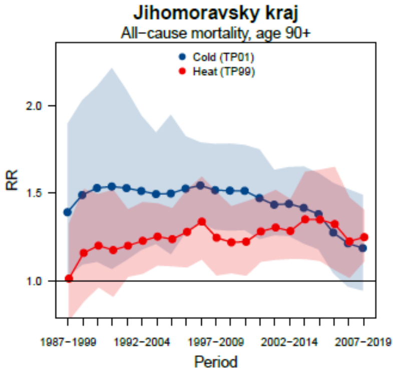
Jihomoravsky kraj
All−cause mortality, age 90+
Cold (TP01)
Heat (TP99)
RR
2.0
1.5
1.0
1987−1999
1992−2004
1997−2009
2002−2014
2007−2019
Period

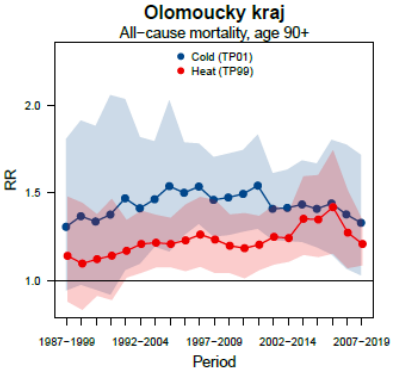
Olomoucky kraj
All−cause mortality, age 90+
Cold (TP01)
Heat (TP99)
RR
2.0
1.5
1.0
1987−1999
1992−2004
1997−2009
2002−2014
2007−2019
Period

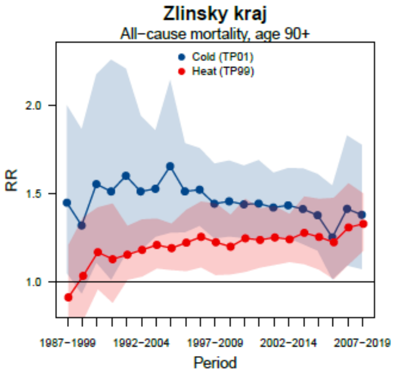
Zlinsky kraj
All−cause mortality, age 90+
Cold (TP01)
Heat (TP99)
RR
2.0
1.5
1.0
1987−1999
1992−2004
1997−2009
2002−2014
2007−2019
Period

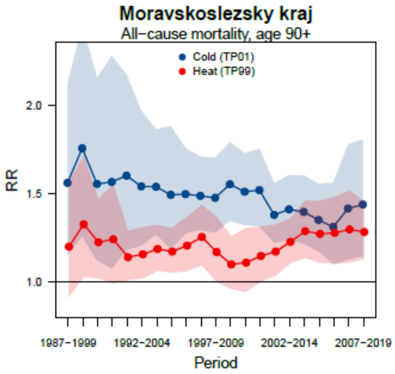
Moravskoslezsky kraj
All−cause mortality, age 90+
Cold (TP01)
Heat (TP99)
RR
2.0
1.5
1.0
1987−1999
1992−2004
1997−2009
2002−2014
2007−2019
Period

Supplementary Figure S16 Trend in RR of death from cardiovascular diseases at 1st (cold) and 99th (heat) temperature percentile among overall population in the regions of the Czech Republic from model with subsets of 13-year moving periods. RR=relative risk, TP01=temperature percentile 1, TP99=temperature percentile 99.

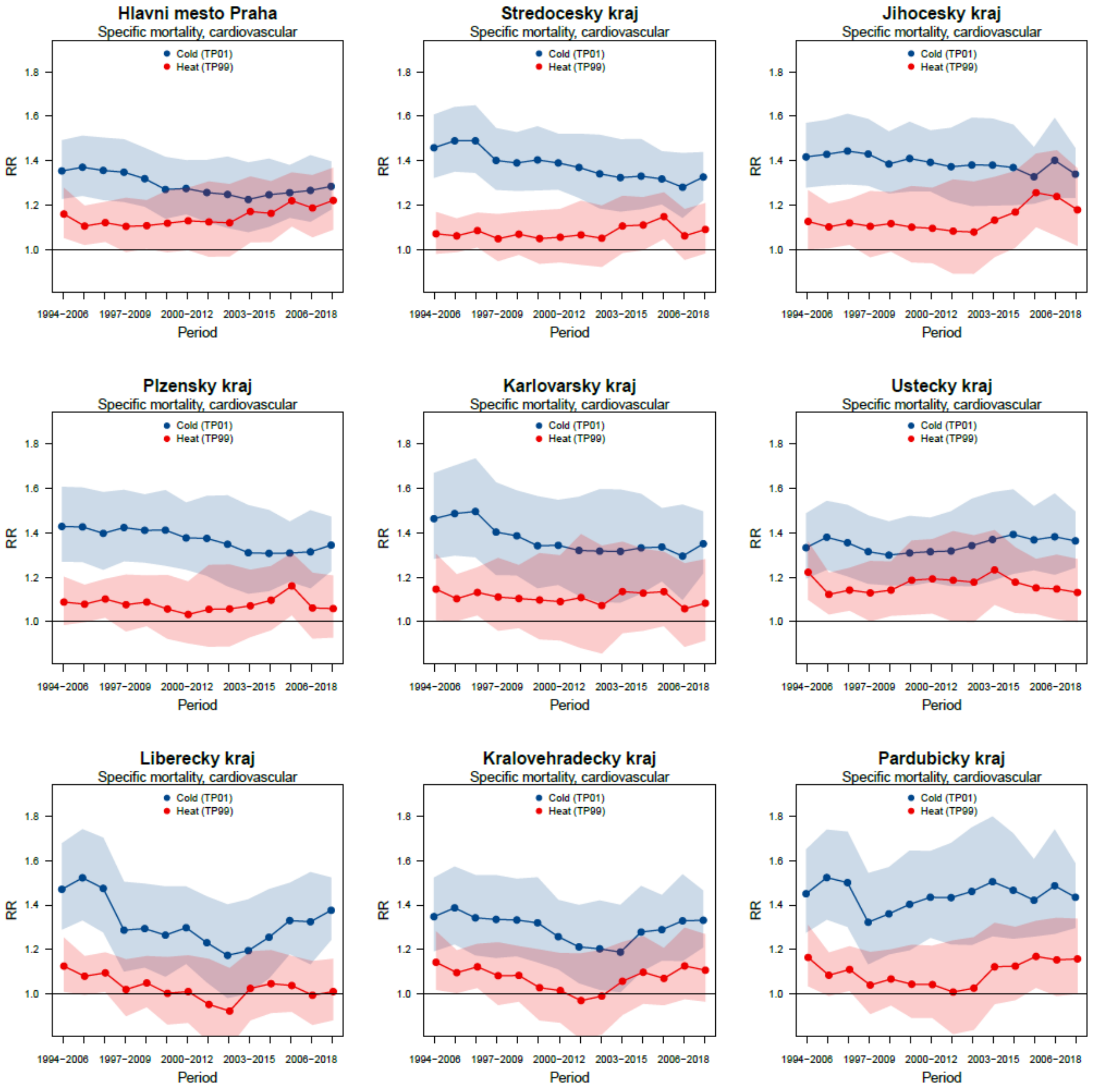

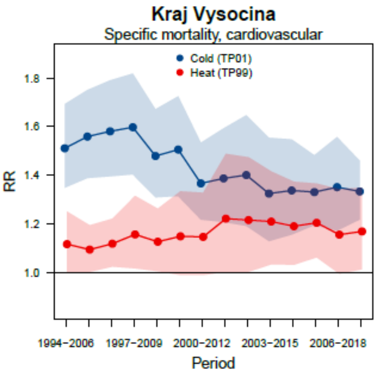
Kraj Vysocina
Specific mortality, cardiovascular
Cold (TP01)
Heat (TP99)
RR
1.0
1.2
1.4
1.6
1.8
1994–2006
1997–2009
2000–2012
2003–2015
2006–2018
Period

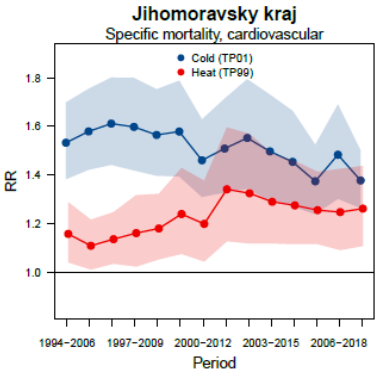
Jihomoravsky kraj
Specific mortality, cardiovascular
Cold (TP01)
Heat (TP99)
RR
1.0
1.2
1.4
1.6
1.8
1994–2006
1997–2009
2000–2012
2003–2015
2006–2018
Period

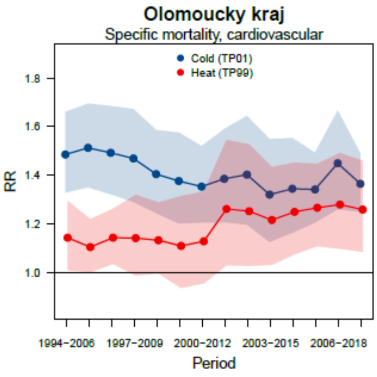
Olomoucky kraj
Specific mortality, cardiovascular
Cold (TP01)
Heat (TP99)
RR
1.0
1.2
1.4
1.6
1.8
1994–2006
1997–2009
2000–2012
2003–2015
2006–2018
Period

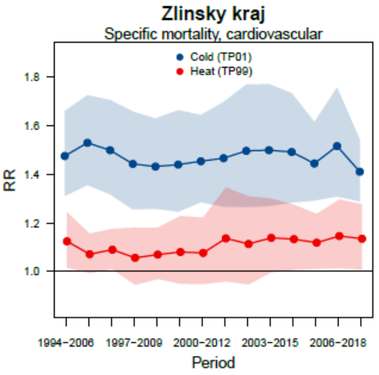
Zlinsky kraj
Specific mortality, cardiovascular
Cold (TP01)
Heat (TP99)
RR
1.0
1.2
1.4
1.6
1.8
1994–2006
1997–2009
2000–2012
2003–2015
2006–2018
Period

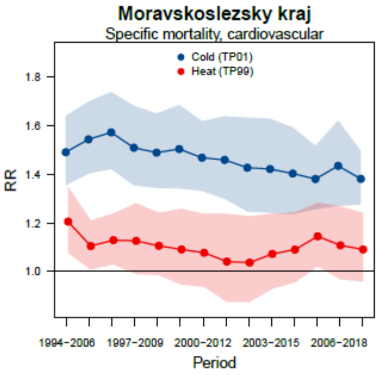
Moravskoslezsky kraj
Specific mortality, cardiovascular
Cold (TP01)
Heat (TP99)
RR
1.0
1.2
1.4
1.6
1.8
1994–2006
1997–2009
2000–2012
2003–2015
2006–2018
Period

Supplementary Figure S17 Trend in RR of death from respiratory diseases at 1st (cold) and 99th (heat) temperature percentile among overall population in the regions of the Czech Republic from model with subsets of 13-year moving periods. RR=relative risk, TP01=temperature percentile 1, TP99=temperature percentile 99.

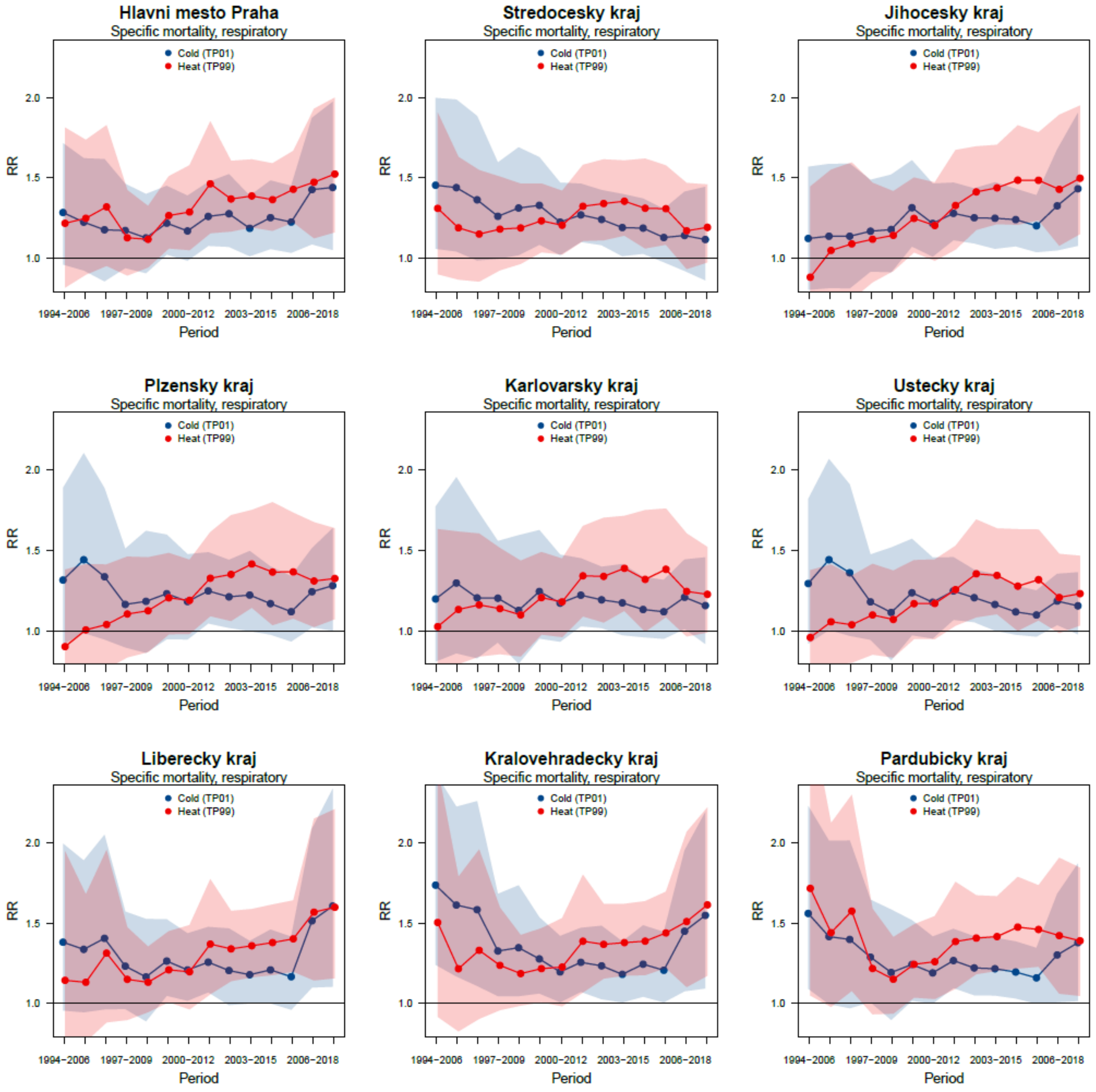

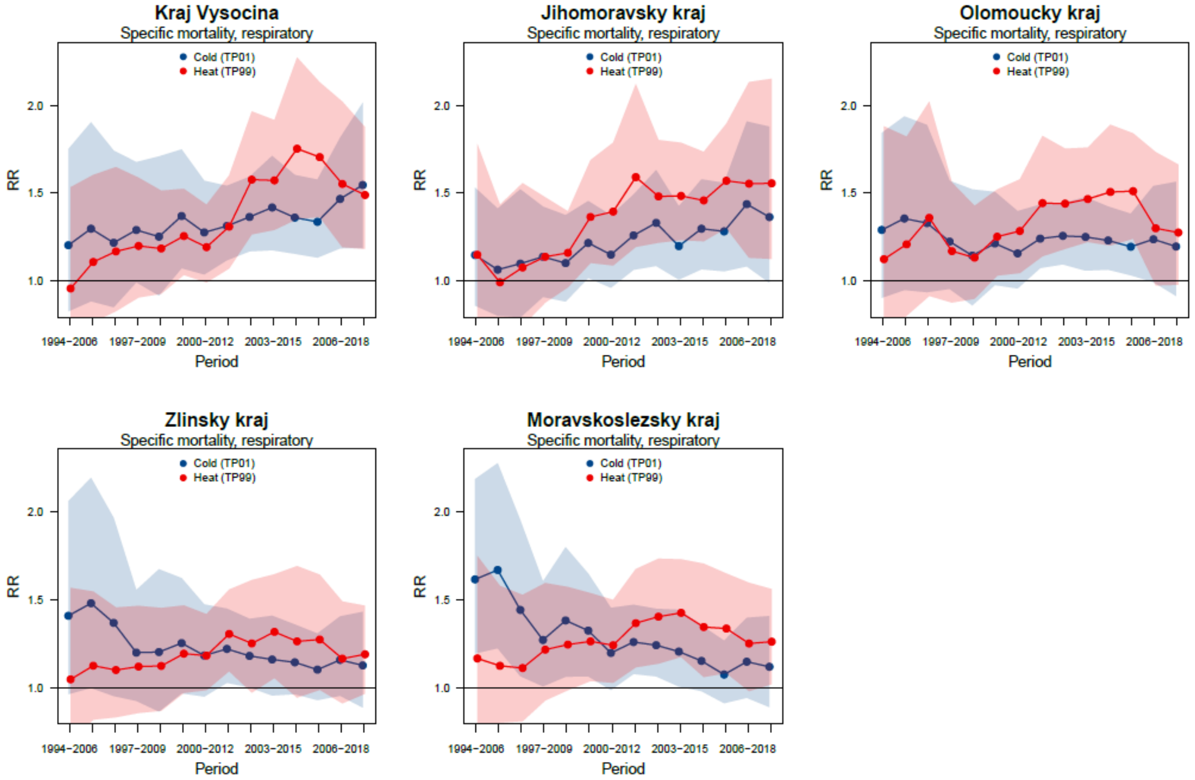
Kraj Vysocina
Specific mortality, respiratory
Jihomoravsky kraj
Specific mortality, respiratory
Olomoucky kraj
Specific mortality, respiratory
Zlinsky kraj
Specific mortality, respiratory
Moravskoslezsky kraj
Specific mortality, respiratory
Cold (TP01)
Heat (TP99)
RR
2.0
1.5
1.0
1994–2006
1997–2009
2000–2012
2003–2015
2006–2018
Period

Supplementary Table S5 Sensitivity analysis by varying modelling choices – Attributable fraction (%) for total, cold and heat temperatures for whole study period and overall population for different parameters in the fitting of the temperature-mortality relationship (95% empirical CI).

**Model**	**Total**	**Cold**	**Heat**
Main model	8.35% (7.22-9.48)	7.99% (6.72-9.01)	0.36% (0.29-0.42)
Df/year for seasonal control: 8	8.47% (8.03-8.89)	8.04% (7.60-8.46)	0.44% (0.36-0.50)
Df/year for seasonal control: 10	7.79% (6.60-8.84)	7.37% (6.15-8.37)	0.42% (0.33-0.49)
Knots for exposure-response: 25th and 75th	6.57% (6.14-7.02)	6.27% (5.81-6.72)	0.31% (0.21-0.39)
Knots for exposure-response: 75th	4.68% (4.25-5.07)	4.52% (4.07-4.95)	0.17% (0.06-0.27)
Maximum lag: 14 days	6.00% (5.57-6.45)	5.50% (5.04-5.93)	0.50% (0.44-0.55)
Maximum lag: 28 days	8.91% (7.40-10.3)	8.60% (6.99-9.89)	0.31% (0.21-0.39)

Cold and heat defined as temperatures lower than MMT and higher than MMT respectively.
